# WGCNA reveals dose-dependent responses and molecular regulatory networks of strigolactone-mediated drought mitigation in *Astragalus membranaceus* var. *mongholicus*

**DOI:** 10.3389/fpls.2026.1811607

**Published:** 2026-05-13

**Authors:** Meng Meng, Huiyu Zhang, Yang Cao, Jiuxuan Zhang, Yiying Liu, Yawen Qi, Jiaxin Liang, Xiaoting Zhai

**Affiliations:** 1 Smart Agriculture Laboratory, College of Information Science and Engineering, Hebei North University, Zhangjiakou, China; 2 Medicinal Resources Laboratory, College of Pharmacy, Inner Mongolia Medical University, Hohhot, China; 3 National Key Laboratory of Crop Improvement and Regulation in North China, College of Horticulture, Hebei Agricultural University, Baoding, China

**Keywords:** *Astragalus membranaceus* var. *mongholicus*, drought stress, ribosome biogenesis, strigolactone, WGCNA

## Abstract

*Astragalus* (*Astragalus membranaceus* var. *mongholicus*) is an economically vital medicinal plant whose productivity is severely hindered by drought. This study investigated the concentration-dependent effects of strigolactone (SL; 0.1, 1, and 10 μmol·L^-1^) on drought-stressed seedlings through integrated physiological and transcriptomic analyses. Results showed that drought induced oxidative damage and inhibited photosynthesis, while SL exhibited distinct concentration-dependent associations with stress mitigation. The 0.1 μmol·L^-1^ concentration yielded weak effects due to insufficient signal intensity, whereas the high concentration of 10 μmol·L^-1^ strengthened osmotic protection associated with the Purple module but coincided with restricted rapid regeneration, paralleling a suppression of soluble protein content and ribosome biogenesis. In contrast, 1 μmol·L^-1^ SL was identified as the appropriate concentration correlating with significantly restored biomass and photosynthetic efficiency. At the molecular level, this recovery is highly associated with the coordinated induction of the Turquoise and Brown modules, which are characterized by the transcriptomic reconstruction of the protein translation machinery and the restoration of hormone signaling. This molecular shift aligns with a hub transcription factor cascade involving 15 hub transcription factors (notably from the WRKY, NAC, and bZIP families) that correlates with the up-regulation of “Ribosome biogenesis” and “Protein processing” pathways, as well as specific downstream functional genes. This coordinated network correlates with a transition toward a functional repair and growth state, although the observed up-regulation of ribosomal genes could also emerge as a secondary consequence of the plant’s overall improved physiological vigor rather than the primary driver. These findings provide a theoretical framework for the high-quality cultivation of *Astragalus* in arid environments.

## 1 Introduction

In the context of global climate change, abiotic stress has emerged as the primary constraint limiting global agricultural productivity and ecosystem stability (Emami Bistgani et al., 2024; Zhi et al., 2024). Among these, drought stress is recognized as the most challenging environmental adversity affecting plant growth and crop yield due to its high frequency, prolonged duration, and extensive coverage (Chen et al., 2024b; Wang et al., 2025c). Water deficit not only directly results in a sharp decline in plant biomass but also severely threatens global food security and the stable supply of high-value medicinal plant resources by disrupting physiological and metabolic equilibrium (Emami Bistgani et al., 2024; Wang et al., 2025g). Recent studies have indicated that drought-induced oxidative damage and metabolic arrest are core factors leading to growth inhibition, a phenomenon confirmed in various crops and medicinal plants (Zhang et al., 2024; Zhi et al., 2024). To rapidly mitigate these devastating impacts and safeguard the accumulation of active medicinal components, relying solely on natural stress adaptation or traditional breeding is often insufficient. Therefore, deeply exploring the molecular mechanisms of plant drought resistance and developing efficient chemical regulation strategies have become urgent tasks in current plant physiology and ecological cultivation research of Chinese medicinal materials (Jian et al., 2022; Wang et al., 2025c).

Plants have evolved a suite of complex mechanisms over long-term evolution to cope with drought environments. At the physiological level, water deficit typically leads to stomatal closure to minimize transpiration, which simultaneously inhibits carbon dioxide fixation, resulting in reduced photosynthetic rates and abnormal chlorophyll fluorescence parameters (Chen et al., 2024b; Wang et al., 2025g). At the biochemical level, the excessive accumulation of reactive oxygen species (ROS) induced by drought leads to severe membrane lipid peroxidation, manifested by a significant increase in malondialdehyde (MDA) content, which further damages the integrity of proteins, nucleic acids, and biological membranes (Chen et al., 2024b; Zhi et al., 2024). Although plants can defend themselves by synthesizing osmotic adjustment substances and activating antioxidant enzyme systems, this endogenous regulatory capacity is often insufficient to maintain normal physiological functions under extreme or persistent drought (Emami Bistgani et al., 2024; Wang et al., 2025c).

Strigolactone (SL) is a class of plant hormones derived from carotenoids that play crucial roles in regulating plant branching, root development, and enhancing plant adaptability to environmental changes (Wang et al., 2024; Dong et al., 2025). Extensive research has explored the application of exogenous SL in enhancing plant drought resistance. During periods of drought, treatment with SL has been shown to significantly enhance photosynthetic efficiency and prevent chlorophyll degradation in *Triticum aestivum* (Song et al., 2023). In *Capsicum chinense*, SL facilitates antioxidant defense mechanisms under water stress by decreasing malondialdehyde (MDA) content and activating antioxidant enzymes (Shu et al., 2023). Furthermore, in *Nicotiana tabacum*, SL effectively modulates the balance of endogenous hormones, including auxin, gibberellin, and abscisic acid, thereby bolstering stress resistance (Wang et al., 2025f). Furthermore, SL can enhance drought tolerance by regulating endogenous abscisic acid (ABA) content, protecting chloroplast ultrastructure, and inducing the expression of stress-resistance genes (e.g., *GhMAX3/4b* or *GmPP2C56*) (Chen et al., 2024a; Dong et al., 2024; Ahmad et al., 2025). Multi-omics analyses have shown that SL is involved in metabolic reconstruction processes including arachidonic acid and linoleic acid metabolism, and can regulate cuticular wax deposition in barley to reduce water loss (Daszkowska-Golec et al., 2023; Cao et al., 2024). SL can also finely tune stomatal morphological characteristics (e.g., stomatal width and density) to cope with extreme environments through interactions with other hormones such as cytokinin and jasmonic acid (Omoarelojie et al., 2019; Ahmad et al., 2025). While extensive research highlights the protective roles of SL in field crops, its application in medicinal plants is just beginning to emerge. Unlike conventional crops, the economic value of medicinal plants relies heavily on the synthesis of pharmacologically active secondary metabolites, which are highly sensitive to stress-induced metabolic disruption. Recent evidence suggests that SL possesses the potential to modulate these specialized secondary metabolic networks to maintain both biomass and medicinal quality under abiotic stress. For instance, exogenous application of SL has been shown to enhance the accumulation of artemisinin in *Artemisia annua* by improving glandular trichome attributes and modulating antioxidant enzyme systems under heavy metal stress (Wani et al., 2023). Similarly, emerging studies on other valuable medicinal plants such as *Salvia miltiorrhiza* and *Mentha* indicate that SL can effectively regulate the biosynthesis of key secondary metabolites including flavonoids and terpenoids, thereby bolstering their pharmacological value under adverse environmental conditions (Li et al., 2024a; Jariani et al., 2024). Despite this immense potential, our overall understanding of SL-mediated drought resistance mechanisms remains limited. Specifically, for *Astragalus*, a legume species that is sensitive to stress and possesses high medicinal value, the specific impacts of SL on morphological repair, physiological reconstruction, and secondary metabolite accumulation during drought stress have not been systematically investigated.

*Astragalus*, a perennial legume widely distributed in arid and semi-arid regions (Ji et al., 2023; Li et al., 2025; Sheng et al., 2024), is an economically vital medicinal plant with high economic and clinical value due to its abundance of bioactive components such as flavonoids, polysaccharides, and triterpene saponins (Dong et al., 2023; He et al., 2024; Wang et al., 2025a). Studies have shown that core components such as *Astragalus* polysaccharides (APS) exhibit significant multi-target pharmacological activities in preventing diabetes, improving neurodegenerative diseases, and assisting in liver cancer immunotherapy (Liu et al., 2024; Shi and Ma, 2024; Wang et al., 2024; Wang et al., 2025e). However, against the background of global climate change, frequent water deficit and temperature fluctuations have become the primary bottlenecks limiting the standardized production of *Astragalus* (Zou et al., 2025). Environmental stress not only significantly inhibits seedling morphological development and biomass accumulation (Li et al., 2024b; Ziaei et al., 2025) but also severely affects the spatio-temporal dynamic accumulation of key active ingredients, such as astragaloside IV and calycosin-7-*O*-β-D-glucoside, by interfering with secondary metabolic networks (Guo et al., 2024; Li et al., 2025), thereby influencing the final quality and clinical efficacy of the medicinal materials (Li et al., 2023; Kwak et al., 2024). Although the importance of SL in regulating plant branching and responding to abiotic stress has been recognized, current research predominantly focuses on routine physiological regulation in model crops and the stress-response regulation of WRKY transcription factors (Wang et al., 2025b; Zhang et al., 2025). For medicinal plants such as *Astragalus*, which are stress-sensitive and rely on specific secondary metabolic networks to sustain quality, the underlying regulatory mechanisms by which SL mediates functional reconstruction of damaged tissues, particularly regarding how transcriptome-metabolome crosstalk is associated with the recovery of protein translation machinery and metabolic networks, remain largely elusive (Li et al., 2023; Liu et al., 2025b). This study systematically analyzed the regulatory network of SL in mitigating drought damage to *Astragalus* via morphological and physiological trait detection combined with whole-transcriptome sequencing integrated with the WGCNA method. We focused on identifying core gene clusters associated with protein repair and the biosynthesis of key secondary metabolites, aiming to elucidate the underlying logic of SL-induced stress reversal in medicinal plants and lay a theoretical foundation for the high-quality cultivation and quality improvement of *Astragalus* in arid regions.

## 2 Materials and methods

### 2.1 *Astragalus* materials, and drought treatment, and SL treatments

Seeds of *Astragalus membranaceus* var. *mongholicus* were obtained from a high-quality standardized cultivation demonstration base. Exogenous rac-GR24 (SL analog, CAS number: 76974-79-3, catalog number: S4840) was purchased from Beijing Solarbio Science & Technology Co., Ltd. (Beijing, China).

The experiments were conducted in an artificial climate chamber at the Experimental Station of Hebei North University (114°53′E, 40°45′N). Environmental conditions were maintained at a 13-h photoperiod, 55% ± 5% relative humidity, and a 25°C/20°C (day/night) temperature cycle. The photosynthetic photon flux density (PPFD) was set at 1000 ± 46 μmol·m^−2^·s^−1^. Regarding plant cultivation, the *Astragalus* seeds were initially germinated in Petri dishes, and uniform seedlings were subsequently transplanted into PVC pots (8 cm×8 cm×8.3 cm) 12 days after germination. Each pot was filled with 120 g of a substrate mixture composed of peat soil (Pinstrup, Denmark) and vermiculite (Shijiazhuang, China) at a volume ratio of 7:3. Throughout the growth phase, the seedlings were watered every two days. To ensure experimental uniformity, four seedlings with consistent growth vigor were retained in each pot during the true leaf stage. Following emergence, standard management measures were implemented, and the field capacity was maintained at approximately 75%. A single-factor experimental design was employed with three biological replicates per treatment, encompassing five distinct treatments: CK (Control, normal conditions), DS (drought with the application of purified water), DS + SL0.1 (drought with the application of 0.1 μmol·L^-1^ strigolactone, SL), DS + SL1 (drought with the application of 1 μmol·L^-1^ SL), and DS + SL10 (drought with the application of 10 μmol·L^-1^ SL). To accurately determine the appropriate SL concentration and investigate its dose-dependent mechanisms, a preliminary screening experiment was conducted using a finer gradient of SL (0.1, 0.5, 1, 5, and 10 μmol·L^-1^). The preliminary physiological results demonstrated that the 1 μmol·L^-1^ SL treatment was the most effective in restoring plant height, chlorophyll content, and photosynthetic parameters (e.g., *Fv/Fm* and Pn) under drought stress, exhibiting a clear peak in efficacy (**Supplementary Figure 1**). Based on this empirical evidence, and supported by previous studies (Chen et al., 2024a; Cao et al., 2024), we selected 0.1, 1, and 10 μmol·L^-1^ to respectively represent low (sub-optimal), optimal, and high (supra-optimal) concentrations for the subsequent comprehensive transcriptomic and physiological analyses. Drought resistance treatments commenced when the plants developed their seventh set of true compound leaves (approximately 21 days after emergence). At this time, water was strictly controlled to reduce soil moisture from 75% field capacity to a target stress level of 30%–35%, which was maintained throughout the drought period. Soil humidity was monitored daily using a LANENDE system soil tester (Shandong, China), and foliar spraying of SL (or purified water for the DS group) began on the same day as drought induction. Spraying was performed four times in total, with a two-day interval between applications, and carried out uniformly at 16:00 until the leaves were wet to the point of slight dripping.

### 2.2 Determination of physiological and biochemical parameters

On the 12th day of drought stress, three *Astragalus* plants per treatment with similar growth vigor were selected to measure plant height (PH) and stem diameter (SD), with fresh apical leaves harvested and immediately stored at -80 °C for physiological and transcriptomic characterization. Since the plants were at the early seedling stage where the medicinal root system had not yet fully developed, measuring root metabolites at this phase possessed limited biological and pharmacological significance. Fresh apical leaves acted as the primary active organs responding to foliar SL application and acute drought stress. Consequently, these leaves were collected as the representative tissue for evaluating early physiological defense mechanisms, namely the protective accumulation of total flavonoids and polysaccharides. Relative chlorophyll content (SPAD) was measured at multiple leaf positions using a SPAD-502Plus meter, while photosynthetic gas-exchange parameters (Pn,Gs,Ci,Tr) and chlorophyll fluorescence (*Fv*/*Fm* and Φ_PSII_) were determined between 9:00 and 11:00 a.m. using a LI-6800 Portable Photosynthesis System and a PAM-2100 fluorometer, respectively (Luqman et al., 2023; Dziwulska-Hunek et al., 2025). Antioxidant activities of peroxidase (POD), superoxide dismutase (SOD), and catalase (CAT) were quantified via guaiacol colorimetry, NBT photochemical reduction, and ultraviolet spectrophotometry, with absorbance measured at 470 nm, 560 nm, and 240 nm (Lyu et al., 2023; Huang et al., 2024). Malondialdehyde (MDA) and soluble protein (SPC) contents were determined using the thiobarbituric acid (TBA) colorimetric and Coomassie Brilliant Blue G-250 staining methods, respectively (Han et al., 2022; Zhang et al., 2023). Finally, total flavonoid content (TFC) and total polysaccharide content (TPS) were assessed using the sodium nitrite-aluminum nitrate-sodium hydroxide and phenol-sulfuric acid colorimetric methods (Mohammed et al., 2022; Bibi et al., 2022), with concentrations quantified against rutin and glucose standard curves (Goncharuk et al., 2022; Zhu et al., 2025).

### 2.3 Transcriptome sequencing and hub gene screening

#### 2.3.1 Transcriptome sequencing, quality control, and evaluation

Total RNA was extracted from *Astragalus* leaves using the E.Z.N.A.^®^ Plant RNA Kit (Omega Bio-tek, Norcross, GA, USA). RNA purity and integrity were verified via a NanoDrop 2000 spectrophotometer and an Agilent 2100 Bioanalyzer. For *de novo* transcriptome analysis, mRNA was enriched using Oligo(dT) magnetic beads and fragmented. The cDNA libraries were constructed through double-stranded cDNA synthesis, end-repair, A-tailing, and adapter ligation. Sequencing was performed on the Illumina NovaSeq 6000 platform to generate 150 bp paired-end reads. Raw data were processed using Trimmomatic (Version 0.39, http://www.usadellab.org/cms/?page=trimmomatic) to remove adapters and low-quality sequences, obtaining clean reads for subsequent assembly (Upton et al., 2023).

#### 2.3.2 *De novo* assembly and functional annotation

The clean reads were *de novo* assembled into a reference transcriptome using Trinity (v1.0, https://github.com/trinityrnaseq/trinityrnaseq/wiki) with default parameters. To reduce redundancy, the assembled transcripts were clustered into a non-redundant unigene set. The quality of the assembly was evaluated based on N50 length and mean length. Functional annotation was then conducted by aligning unigenes against the GO and KEGG databases using BLASTx.

#### 2.3.3 Quantification and differential expression analysis

Clean reads were mapped to the assembled non-redundant unigene set using Bowtie2 (version 2.2.6, http://bowtie-bio.sourceforge.net/bowtie2/index.shtml), and gene expression levels were quantified using the FPKM method. The FPKM value was calculated based on the following formula:


$$FPKM=\frac{mapped\;fragments\;of\;transcript}{Total\;Count\;of\;mapped\;fragments(Millions)\times Length\;of\;transcript(kb)}$$

In this formula: mapped fragments of transcript refers to the number of fragments (paired-end reads) mapped to a specific transcript; Total Count of mapped fragments (Millions) is the total count of mapped fragments across the transcriptome, normalized by 106; and Length of transcript (kb) represents the transcript length in kilobases (103 bp).

Differential expression analysis between groups was performed using the DESeq2 R package (version 1.44.0, http://bioconductor.org/packages/stats/bioc/DESeq2/) (Love et al., 2014). Genes with an |log_2_(FoldChange)|≥1 and a false discovery rate (FDR) < 0.01 were identified as differentially expressed genes (DEGs). Functional annotation and enrichment analysis were conducted by aligning DEGs against the GO and KEGG databases. GO terms were categorized into biological processes (BP), molecular functions (MF), and cellular components (CC). KEGG pathway enrichment analysis was employed to identify significantly activated metabolic pathways under drought and SL treatments.

#### 2.3.4 Trend analysis and functional enrichment

To identify gene expression profiles responsive to drought stress and exogenous SL, K-means clustering was performed on the Z-score normalized FPKM values of all DEGs. The optimal number of clusters (k) was determined using the gap statistic. Clusters exhibiting expression patterns consistent with the trends of physiological indicators were selected as representative modules. Subsequently, KEGG pathway enrichment analysis was conducted for each cluster to identify significantly activated metabolic pathways involved in the plant’s response to drought and SL treatments.

#### 2.3.5 WGCNA and module-trait association analysis

Prior to network construction, the initial set of 100,787 unigenes was filtered to eliminate genes exhibiting low expression or low variance across all samples, thereby improving the accuracy of the network. This pre-filtering was conducted using the varFilter function from the genefilter R package with a variance filtering threshold set to 0.5. Following this step, a highly reliable subset of 49,465 genes was retained. Weighted Gene Co-expression Network Analysis (WGCNA) was performed using the WGCNA R package (version 1.70, https://CRAN.R-project.org/package=WGCNA) to construct co-expression modules. To ensure data quality prior to module construction, a sample clustering dendrogram was generated to evaluate the presence of potential outliers. The results confirmed that all samples were clustered appropriately based on their treatment groups, and no outlier samples were removed (**Supplementary Figure 2**). Subsequently, the soft-thresholding power (β) was selected based on the criteria of scale independence and mean connectivity. Specifically, a soft-thresholding power of β = 14 was chosen to ensure the network achieved a scale-free topology fit index of R² > 0.8 (**Supplementary Figure 3**). Core modules were identified by calculating Pearson correlations between module eigengenes (MEs) and the raw values of physiological traits (e.g., antioxidant enzymes and chlorophyll fluorescence). To preserve biological variance and ensure accurate correlation, raw data from all 15 individual biological replicates were directly paired with their corresponding transcriptomic profiles. To narrow down the core candidate genes, transcription factors (TFs) within the significantly enriched pathways of the core modules were intersected with the representative gene clusters identified by K-means clustering. Due to the lack of a specific reference interactome for *A. membranaceus*, the candidate genes were mapped to their orthologs in *Cicer arietinum* (Blastx, E-value < 1e-5), and the resulting gene set was imported into the STRING database(https://string-db.org/) to construct a protein-protein interaction (PPI) network. The network was visualized using Cytoscape (version 3.9.1, https://cytoscape.org/), and hub genes were finally identified based on their connectivity (degree) scores. For better visualization and interpretation, the identified hub genes were assigned putative names (e.g., *AmWRKY40*) based on their *C. arietinum* orthologs or functional annotations. Detailed information regarding the correspondence between Transcript IDs, orthologous IDs, and assigned gene symbols is provided in **Supplementary Table 1**.

#### 2.3.6 qRT-PCR validation and functional verification

To validate the RNA-seq data, qRT-PCR was conducted on 12 genes randomly selected for expression analysis. Gene-specific primers were designed using Primer Premier 5 software (Premier Biosoft International, http://www.premierbiosoft.com/), and the detailed primer sequences are listed in **Supplementary Table 2**. Total RNA of high integrity was isolated from *Astragalus* samples and subsequently reverse-transcribed into cDNA using the HiFiscript cDNA Synthesis Kit (CWBIO, Beijing, China) following the manufacturer’s instructions. qRT-PCR program was run in a Bio-Rad iQ5 Thermo Cycler (Bio-Rad, Hercules, CA, USA) using 2 × Fast Super Evagreen qPCR mastermix (US Everbright Inc., Daly City, CA, USA). The cycling program consisted of: 95 °C for 3 min; followed by 40 cycles of 95 °C for 10 s, 60 °C for 30 s. Each reaction was performed in three biological replicates, with three technical replicates per biological sample to ensure reproducibility. Fluorescence intensity was recorded in real time and was directly proportional to the amount of amplified DNA. A stable reference gene *18S rRNA* was used as an internal control for data normalization (Upton et al., 2023). The relative mRNA abundance for each gene was determined by the $2^{-\Delta \Delta C_{T}}$ method (Wang et al., 2025c). The calculation formula was as follows:


$$\Delta C_{T}=C_{T,target}-C_{T,18S}$$

$$\Delta \Delta C_{T}=\;\Delta C_{T,treatment}-\Delta C_{T,control}$$

$$Relative\;expression=\;2^{-\Delta \Delta C_{T}}$$

### 2.4 Statistical analysis

Statistical analysis was performed using SPSS 26.0 (IBM, https://www.ibm.com). All experimental data, including agronomic traits, photosynthetic parameters, and biochemical indicators, were obtained from at least three independent biological replicates and are presented as the mean ± standard error (SE). Before formal analysis, the normality and homogeneity of variance were verified using the Shapiro-Wilk test and Levene’s test, respectively. The effects of drought stress and different concentrations of exogenous SL were evaluated by one-way analysis of variance (ANOVA), followed by least significant difference (LSD) *post hoc* tests for multiple comparisons. Statistical significance was set at *p* < 0.05. For transcriptome data, sample consistency and reliability were assessed via Pearson correlation coefficients and principal component analysis (PCA). Scientific figures and heatmaps were generated using GraphPad Prism 9.0 (https://www.graphpad.com/), and the R programming language.

## 3 Results

### 3.1 Phenotypic and physiological responses of *Astragalus* under drought stress across treatments

**Figure 1:**
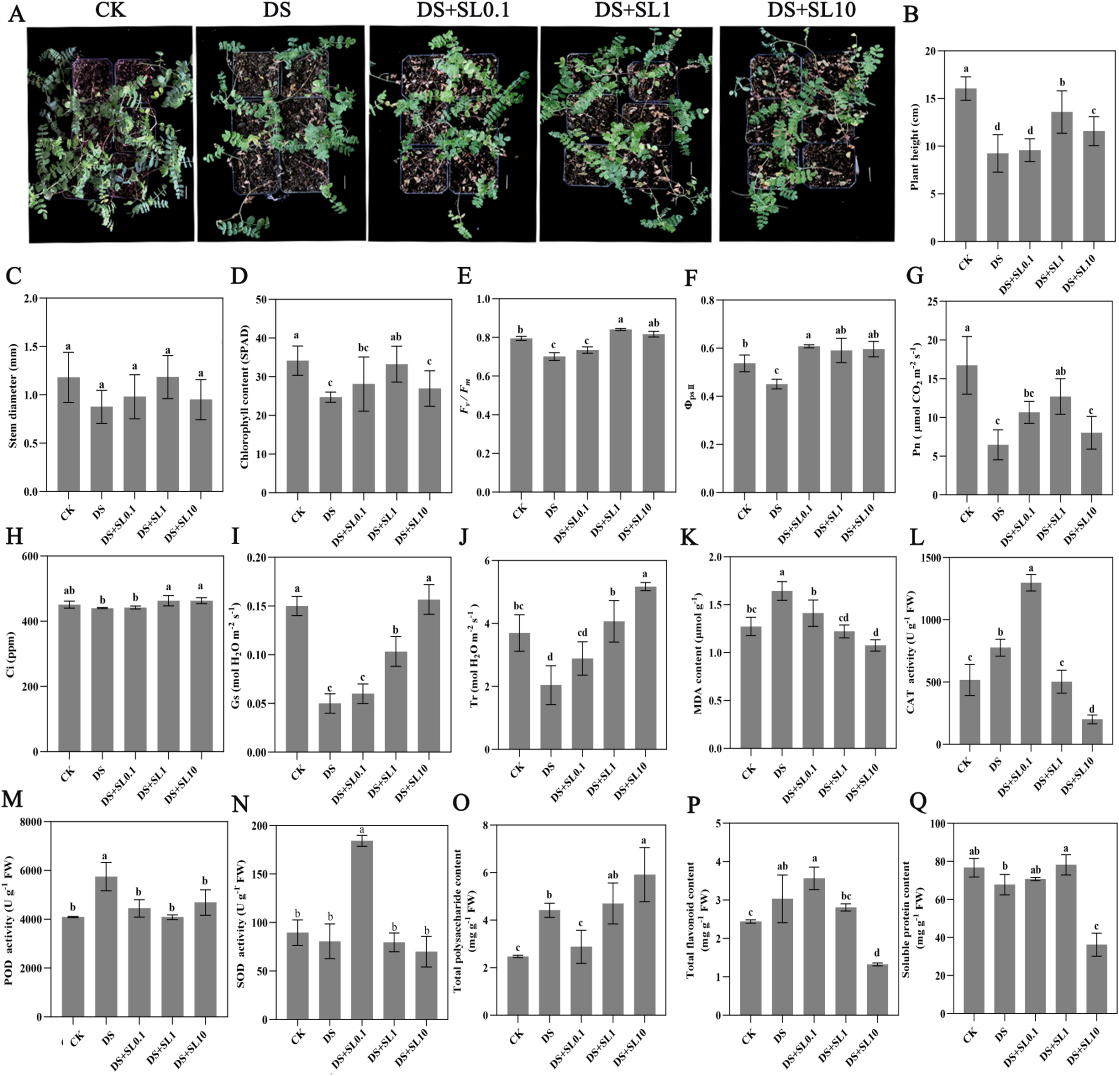
Physiological and phenotypic responses of *Astragalus* plants under different treatments.**(A)** Phenotypes of *Astragalus* plants in CK (well-watered control), DS (drought stress), DS+SL0.1, DS+SL1, and DS+SL10 (drought stress supplemented with 0.1, 1, and 10 μmol·L^-1^ exogenous SL, respectively) groups, alongside histograms depicting various physiological indices of *Astragalus* plants in each group; **(B)** Plant height (PH); **(C)** Stem diameter (SD); **(D)** Chlorophyll content (SPAD); **(E)** Maximum photochemical efficiency of PSII (*F_v_/F_m_*); **(F)** Actual photochemical efficiency of PSII (Φ_PS II_); **(G)** Net photosynthetic rate (Pn); **(H)** Intercellular CO_2_ concentration (Ci); **(I)** Stomatal conductance (Gs); **(J)** Transpiration rate (Tr); **(K)** Malondialdehyde (MDA) content; **(L)** Catalase (CAT) activity; **(M)** Peroxidase (POD) activity; **(N)** Superoxide dismutase (SOD) activity; **(O)** Total polysaccharide content (TPS); **(P)** Total flavonoid content (TFC); **(Q)** Soluble protein content (SPC). Data are presented as means ± SE (n=3). Different lowercase letters indicate significant differences among treatments (LSD test, *p* < 0.05). Scale bar: 2 cm.

Drought stress significantly suppressed the growth of *Astragalus* with PH decreasing from 17.05 to 9.25 cm and impaired photosynthetic efficiency as evidenced by precipitous declines in Pn, SPAD, Fv/Fm, and stomatal parameters alongside a reduction in SPC. Simultaneously, drought stress induced severe lipid peroxidation, as evidenced by a 29.13% increase in MDA. This also triggered a defensive compensatory response, characterized by the significant accumulation of POD and CAT activities, along with increased levels of TPS and TFC (**Figures 1A–Q**). The intervention of exogenous SL demonstrated a pronounced concentration dependent mitigation effect. Specifically, the 0.1 μmol·L^-1^ treatment primarily bolstered antioxidant and secondary metabolic defenses, causing CAT activity to peak at 1296.67 U·g^-1^ FW and TFC to reach 3.56 mg·g^-1^ FW. The 1 μmol·L^-1^ treatment proved to be the most effective for restoring growth and photosynthetic assimilation capacity, where PH, SD, and Gs surpassed those of all other treatment groups while SPC recovered to 78.2 mg·g^-1^ FW. In contrast, the high concentration 10 μmol·L^-1^ treatment reduced MDA to its minimum value of 1.07 μmol·g^-1^ FW and stimulated substantial polysaccharide accumulation to 5.92 mg·g^-1^ FW, which represented a 138.71% increase relative to CK. However, this concentration resulted in a drastic decline in SPC to 36.17 mg·g^-1^ FW, which suggested that this concentration might have an inhibitory impact on protein homeostasis. Ultimately, the application of exogenous SL at a moderate concentration of 1 μmol·L^-1^ was associated with drought stress mitigation, coinciding with the synergistic enhancement of antioxidant defenses, photosynthetic recovery, and improved growth. Conversely, a higher concentration of 10 μmol·L-1 further reduces antioxidant enzyme content and stimulates polysaccharide accumulation but adversely affects protein homeostasis. This illustrates a concentration-dependent dual effect of SL action.

### 3.2 Transcriptomic characteristics of *Astragalus* in response to drought and SL treatments

#### 3.2.1 Quality control of sequencing data and assembly evaluation

To systematically investigate the molecular mechanisms of drought stress in *Astragalus* plants, RNA-seq was performed on leaf samples from five treatment groups (CK, DS, DS+SL0.1, DS+SL1, and DS+SL10). A total of 788,687,578 raw reads were generated across the 15 samples, and following stringent adapter removal and low-quality filtering, 779,650,730 clean reads were obtained, with individual sample outputs ranging from 46.92 to 58.31 million reads. Quality assessment indicated that Q20 base percentages ranged from 99.10% to 99.42%, Q30 base percentages ranged from 96.65% to 97.80%, and GC content varied from 43.48% to 44.76% (**Supplementary Table 3**).

**Figure 2:**
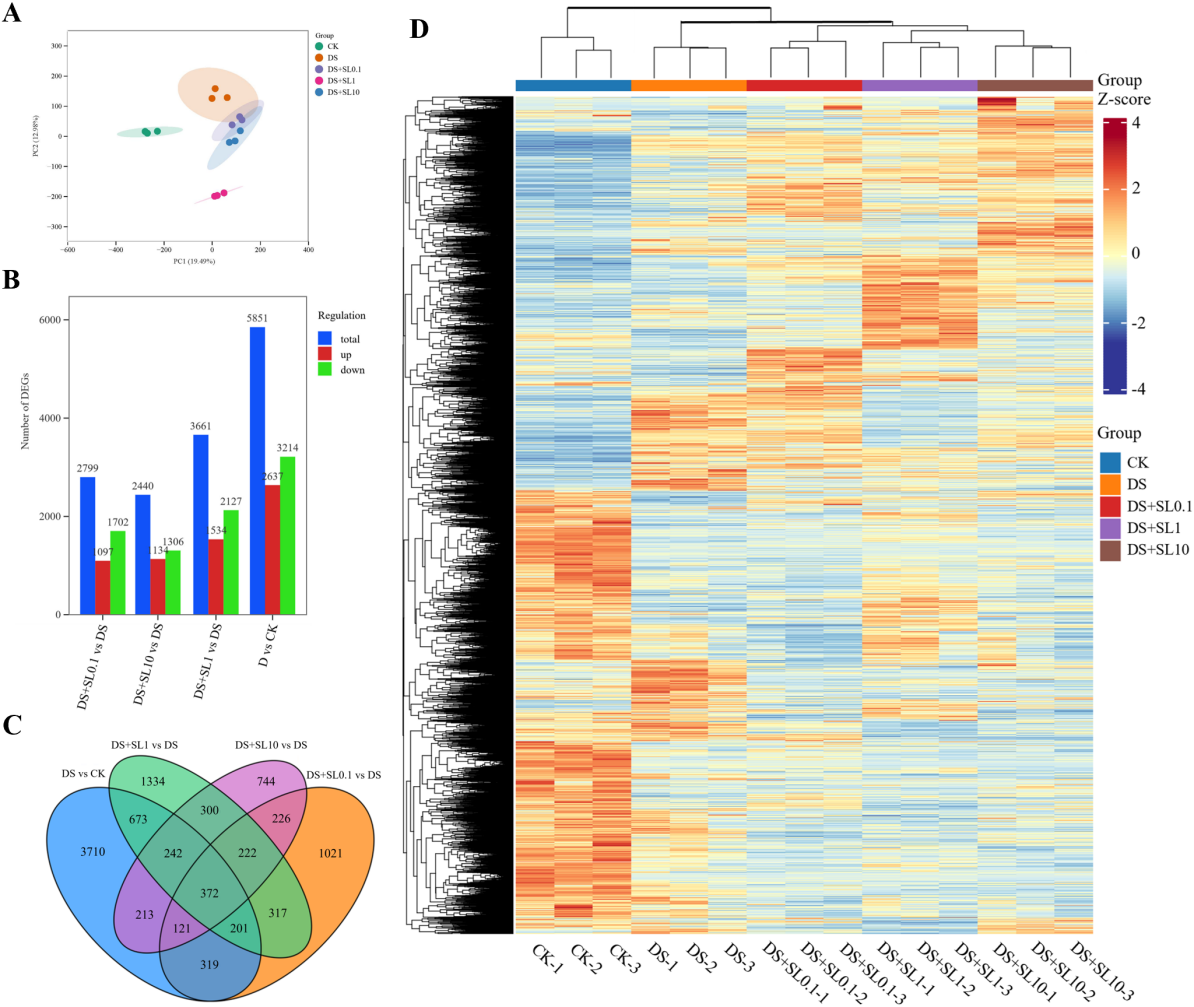
Global analysis of differentially expressed genes (DEGs) in *Astragalus* under different treatments. **(A)** Principal component analysis (PCA) plot of samples across different treatment groups; **(B)** statistics regarding the number of up-regulated and down-regulated DEGs among different comparison groups; **(C)** Venn diagram of DEGs shared across various comparison groups; and **(D)** hierarchical clustering heatmap of DEGs across all samples.

Following *de novo* assembly using Trinity v1.0, 100,787 unigenes and 184,214 transcripts were obtained, with average N50 lengths of 1,781 and 1,618 bp and average lengths of 1,072 and 1,331 bp, respectively. Transcripts within the 200–300 bp range constituted the most abundant category, followed by the 300–400 bp range, together representing the largest proportion of total transcripts (**Supplementary Figure 4**). Pearson correlation coefficients (PCCs) between biological replicates exceeded 0.92 (**Supplementary Figure 5**), and Principal Component Analysis (PCA) demonstrated that the control group (CK) was significantly separated from the various drought treatment groups (**Figure 2A**). Notably, the DS+SL1 group exhibited a unique spatial distribution, confirming the reliability of the biological replicates and the robustness of the treatment effects.

#### 3.2.2 Identification and expression patterns of differentially expressed genes

Significant DEGs across the treatment groups were identified using the criteria of |log_2_​FC|[cites​tart]≥1 and FDR < 0.01. A total of 5,851 DEGs (2,637 up-regulated and 3,214 down-regulated) were identified in the CK vs. DS comparison, revealing a severe perturbation of the transcriptome by drought stress. The application of exogenous SL modified the gene expression profiles in a clear concentration-dependent manner: compared to the DS group, the DS+SL0.1, DS+SL1, and DS+SL10 groups contained 2,799 (1,097 up/1,702 down), 3,661 (1,534 up/2,127 down), and 2,440 (1,134 up/1,308 down) DEGs, respectively (**Figure 2B**). Venn diagram analysis indicated that 372 core DEGs were consistently regulated across the four comparison groups (**Figure 2C**), and hierarchical clustering confirmed the high internal consistency of treatment replicates and distinct differentiation between experimental groups (**Figure 2D**).

#### 3.2.3 GO functional enrichment analysis

**Figure 3:**
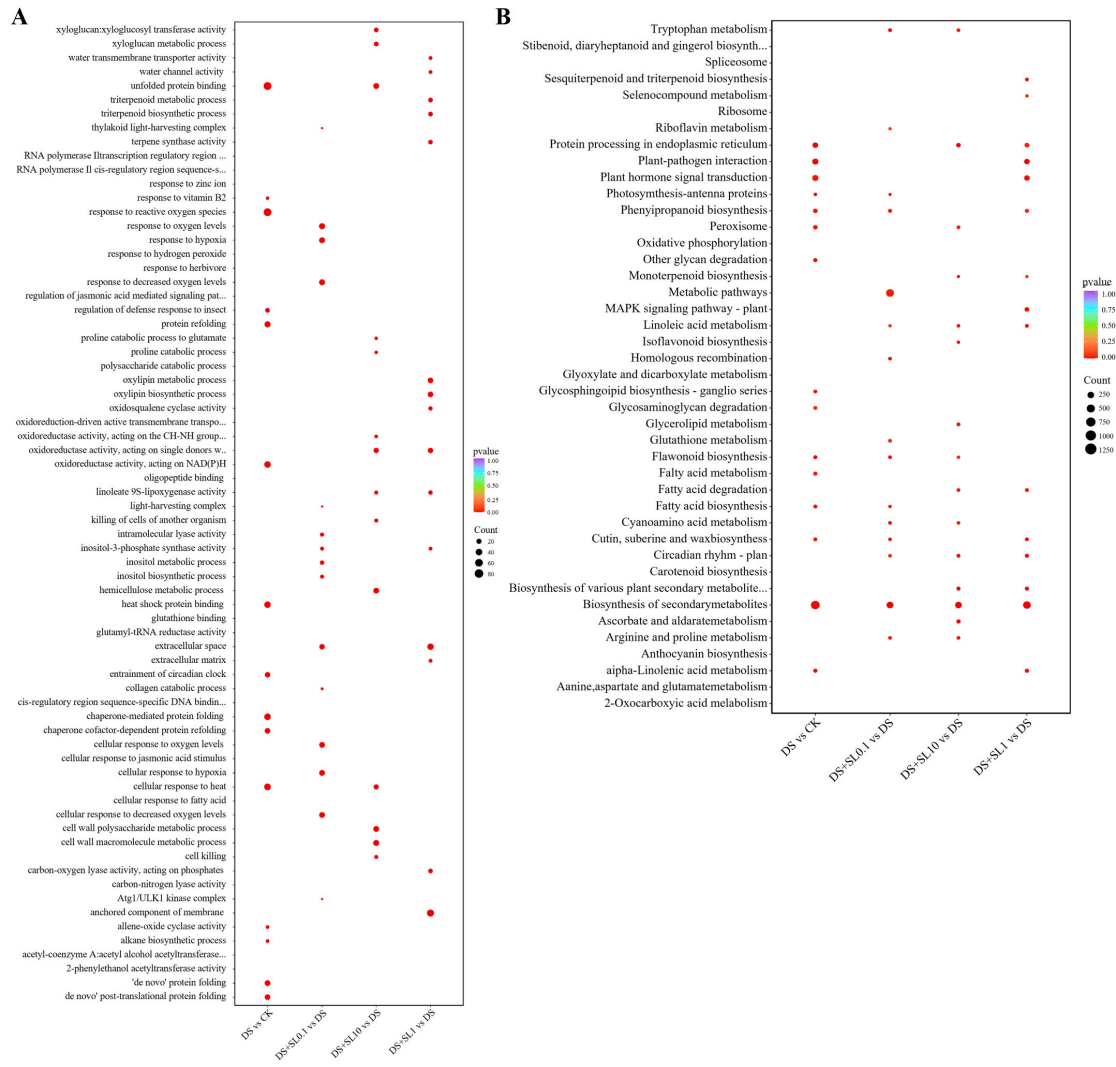
Bubble plot of GO functional and KEGG pathway enrichment analysis of differentially expressed genes (DEGs) in *Astragalus* under different treatments. **(A)** Bubble plot of GO functional enrichment of DEGs in *Astragalus* under different treatments; **(B)** Bubble plot of KEGG pathway enrichment of DEGs in *Astragalus* under different treatments.

GO functional enrichment analysis further clarified the regulatory pathways influenced by drought and SL treatments (**Figure 3A**). Drought stress (CK vs. DS) primarily triggered emergency protective responses, as evidenced by the significant enrichment of terms such as “unfolded protein binding”, “protein folding”, “chaperone-mediated protein folding”, and “response to reactive oxygen species”, reflecting drought-induced protein damage and oxidative stress. Exogenous SL alleviated this damage through distinct biological processes depending on the dosage. The 0.1 μmol·L^-1^ concentration focused on early signaling pathways including “response to oxygen levels” and “response to hypoxia”. The 1 μmol·L^-1^ concentration significantly up-regulated “water channel activity”, “water transmembrane transporter activity” and “triterpenoid biosynthetic process” to enhance water balance and chemical defense. In contrast, the 10 μmol·L^-1^ concentration was enriched in cell wall remodeling terms involving “xyloglucan metabolic process” and “cell wall polysaccharide metabolic process” alongside “proline catabolic process”, which indicated an emphasis on structural reinforcement and fine tuning of osmolyte homeostasis under extreme stress.

#### 3.2.4 KEGG pathway enrichment analysis

To deeply elucidate the molecular pathways of *Astragalus* in response to drought stress and regulation by SL, this study conducted global and directional (up-regulated/down-regulated) KEGG pathway significance enrichment analysis on the differentially expressed genes (DEGs) among various treatment groups. In the DS vs. CK comparison group, drought stress significantly affected the basal metabolism and defense systems of *Astragalus*, primarily involving “Biosynthesis of secondary metabolites” and “Plant hormone signal transduction” (**Figure 3B**), where up-regulated genes were significantly enriched in the “Metabolic pathways” and “Flavonoid biosynthesis” pathways (**Supplementary Figure 6A**), while down-regulated genes were highly concentrated in the “Photosynthesis-antenna proteins” and “Protein processing in endoplasmic reticulum” terms (**Supplementary Figure 6B**), reflecting that water deficiency forced the inhibition of light-harvesting efficiency and led to the obstruction of intracellular protein folding and processing.

Exogenous application of SL at different concentrations exhibited significant directional compensatory effects on the aforementioned metabolic inhibition, showing a clear dose-dependency. At 0.1 μmol·L^-1^ SL (DS+SL0.1 vs. DS), “Metabolic pathways” and “Circadian rhythm - plant” were significantly regulated. Up-regulated genes were significantly enriched in “Flavonoid biosynthesis”, “Linoleic acid metabolism”, and “Circadian rhythm - plant” (**Supplementary Figure 6C**), while down-regulated genes were distributed in pathways such as “Cutin, suberin and wax biosynthesis” and “Starch and sucrose metabolism” (**Supplementary Figure 6D**), indicating that low-concentration SL prioritize the activation of early stress responses and rhythm signal regulation. Under 1 μmol·L^-1^ SL treatment (DS+SL1 vs. DS), “Sesquiterpenoid and triterpenoid biosynthesis” showed significant enrichment signals. Up-regulated genes were highly enriched in “Sesquiterpenoid and triterpenoid biosynthesis” and “Plant pathogen interaction” (**Supplementary Figure 6E**) effectively promoting the accumulation of key bioactive components in *Astragalus* such as triterpenoid saponins and enhancing broad spectrum resistance. Down-regulated genes were primarily enriched in “alpha-Linolenic acid metabolism” and “ABC transporters” (**Supplementary Figure 6F**). Further analysis of the regulatory effects of 10 μmol·L^-1^ SL (DS+SL10 vs. DS) revealed significant regulation of “Isoflavonoid biosynthesis” and “Arginine and proline metabolism” pathways, with up-regulated genes significantly enriched in these two pathways (**Supplementary Figure 6G**). Down-regulated genes were mainly involved in “Protein processing in endoplasmic reticulum” and “Circadian rhythm - plant” (**Supplementary Figure 6H**), suggesting that high-concentration SL may respond to extreme water stress by strengthening the metabolism of osmotic regulators. In summary, the exogenous SL effectively compensated for drought-induced functional inhibition, a process closely associated with the up-regulation of key defense pathways at the transcriptional level, such as flavonoids, triterpenoids, and amino acid metabolism.

#### 3.2.5 Gene expression trend analysis and KEGG functional enrichment

**Figure 4:**
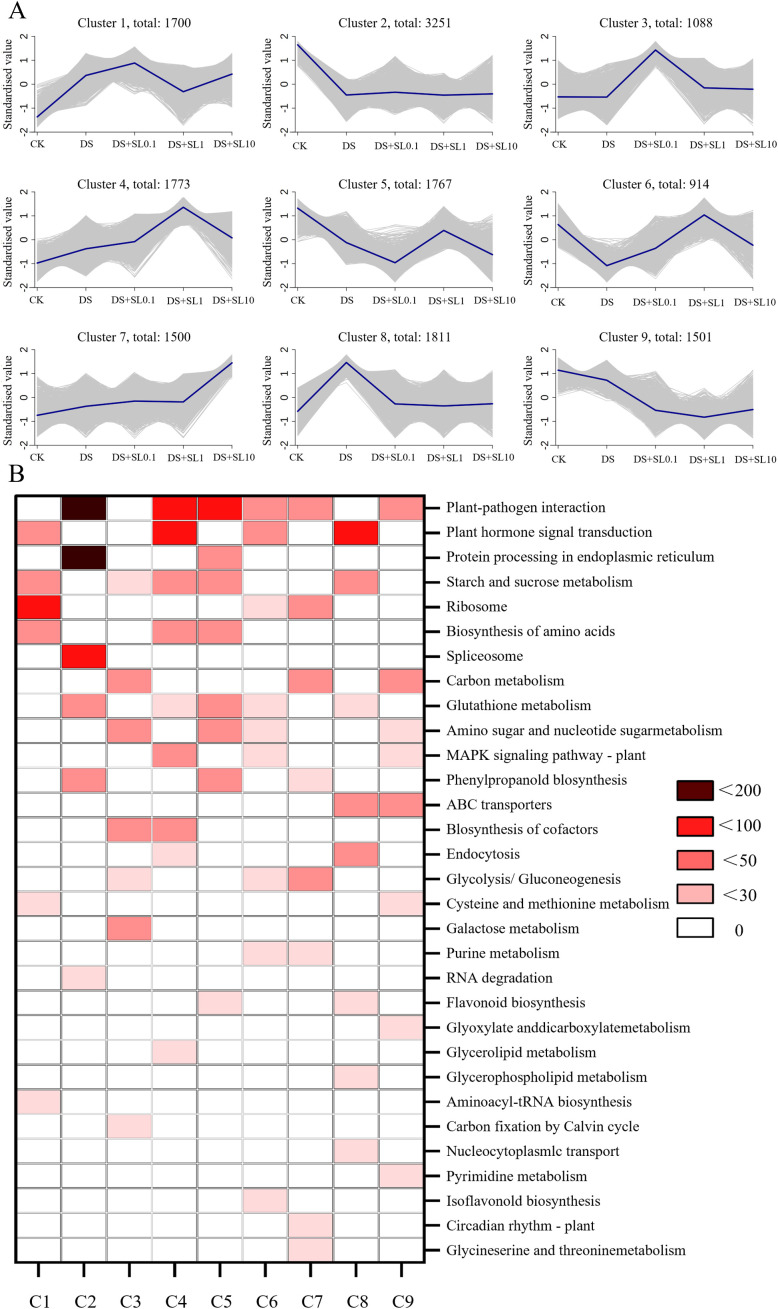
Trend clustering and KEGG enrichment analysis of DEGs under different treatments. **(A)** K-means clustering profiles of DEGs (C1–C9); **(B)** KEGG pathway enrichment heatmap across clusters C1–C9. The color gradient from white to dark red represents increasing enrichment significance, indicated by the number of genes.

To further explore the transcriptional regulatory mechanism of *Astragalus* in response to drought stress, this study utilized the K-means algorithm to perform clustering analysis on the expression kinetics of 15,305 genes, identifying a total of 9 gene clusters (**Figure 4A**). Based on the similarity of expression trends, these gene clusters were categorized into four typical patterns. First, Clusters 2, 5, and 9 were highly expressed in CK group but sharply down-regulated under drought stress and remained at low levels under subsequent SL treatments of varying concentrations. Functional enrichment showed that this pattern primarily involved “Plant-pathogen interaction”, “Protein processing in the endoplasmic reticulum”, “Carbon metabolism”, and “Glutathione metabolism” (**Figure 4B**). Second, Cluster 8 reached its peak in the DS group but rapidly declined after SL treatment, showing significant enrichment in “ABC transporters” and “pyrimidine metabolism” pathways.

Meanwhile, the remaining gene clusters exhibited the positive regulatory characteristics of SL on gene expression. The expression levels of Clusters 1, 3, 4, and 6 were positively up-regulated following SL treatments. The 0.1 μmol·L^-1^ SL concentration was preferentially associated with the up-regulation of Cluster 1 (“Ribosome”, “Biosynthesis of amino acids”) and Cluster 3 (“MAPK signaling pathway”), whereas 1 μmol·L^-1^ SL concentration had the most significant up-regulatory effect on Clusters 4 and 6, mainly involving secondary metabolic pathways such as “Flavonoid biosynthesis”. Additionally, the gene expression level of Cluster 7 steadily increased with the concentration of SL and was significantly enriched in pathways such as the “Spliceosome”, “Endocytosis”, and “Circadian rhythm - plant”.

### 3.3 Construction of the damage-repair co-expression network based on WGCNA

In this study, WGCNA was performed on transcriptomic data comprising 49,465 genes from *Astragalus*. Through module hierarchical clustering and trait-association analysis, six core co-expression modules were systematically identified.

In the CK group, the Blue and Brown modules exhibited high expression abundance. Among them, the Blue module was significantly enriched in “Protein processing in endoplasmic reticulum”, “Photosynthesis-antenna proteins”, “Ubiquitin mediated proteolysis”, “Plant-pathogen interaction”, “Phenylalanine metabolism”, and “Spliceosome” pathways, showing a significant positive correlation with Pn and PH. The Brown module was enriched in “Plant-pathogen interaction”, “Plant hormone signal transduction”, “alpha-Linolenic acid metabolism”, “Peroxisome”, and “Ubiquitin mediated proteolysis” pathways, correlating significantly and positively with PH, SD, SPAD, and Pn; however, both modules exhibited a sharp down-regulation following drought stress. After drought stress, the Black module was significantly up-regulated and enriched in “Valine, leucine, and isoleucine biosynthesis”, “Biosynthesis of secondary metabolites”, “Cyanoamino acid metabolism”, and “Cutin, suberine and wax biosynthesis” pathways, showing significant positive correlations with MDA and POD, and significant negative correlations with PH, SPAD, *Fv*/*Fm*, Φ_PSII_, Pn, and Gs. In the treatment groups subjected to drought stress and 0.1 μmol·L^-1^ SL concentration supplementation, genes in the Magenta module showed obvious up-regulation and were enriched in “Plant hormone signal transduction”, “Riboflavin metabolism”, and “Circadian rhythm - plant” pathways. This module was significantly positively correlated with membrane lipid peroxidation indicators MDA and CAT and significantly negatively correlated with PH, SD, SPAD, *Fv*/*Fm*, Ci, Gs, and Tr. The 1 μmol·L^−1^ SL concentration, on one hand, was specifically associated with the Turquoise module, which was enriched in “Ribosome”, “Proteasome”, “SNARE interactions in vesicular transport”, “Ribosome biogenesis in eukaryotes”, and “RNA degradation”, showing distinct positive correlations with SD, *Fv*/*Fm*, and Ci. On the other hand, it coincided with a rebound of the Brown module, correlating with the recovery of indicators such as PH, SD, SPAD, and Pn. The 10 μmol·L^−1^ SL concentration was significantly associated with the Purple module, which was enriched in “Glyoxylate and dicarboxylate metabolism” and “mRNA surveillance” pathway, showing significant positive correlations with Ci, Tr, and TPS, while being significantly negatively correlated with MDA, CAT, TFC, and SPC.

### 3.4 Identification of hub genes involved in SL-mediated drought stress response

#### 3.4.1 Specific expression patterns and functional associations of the turquoise and brown modules

**Figure 5:**
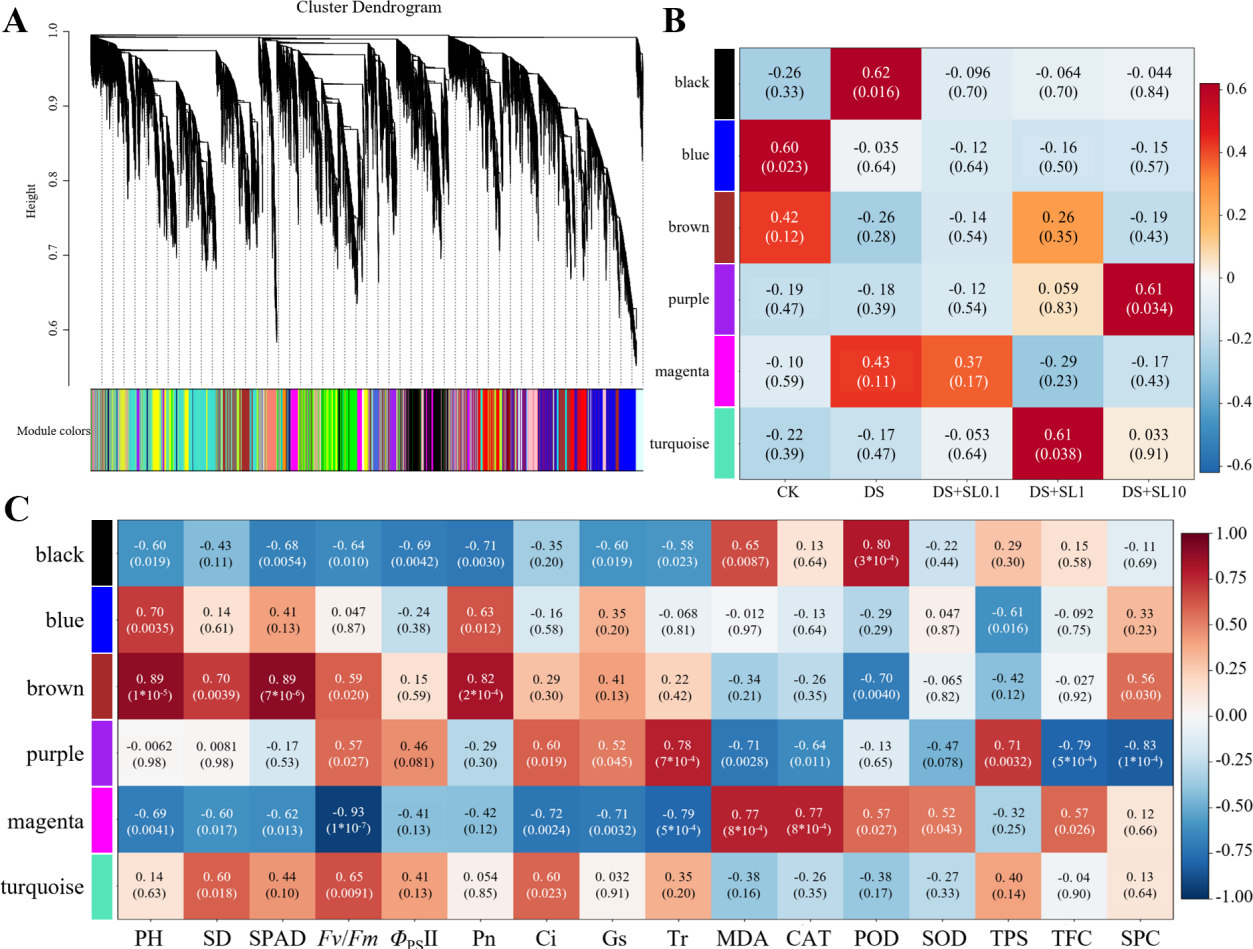
WGCNA and core module correlation evaluation in *Astragalus*. **(A)** Hierarchical clustering dendrogram of modules (soft-thresholding power β=14, R^2^>0.8); **(B)** Module–sample correlation heatmap; **(C)** Module–trait correlation heatmap. Blue and red indicate negative and positive correlations, respectively.

**Figure 6:**
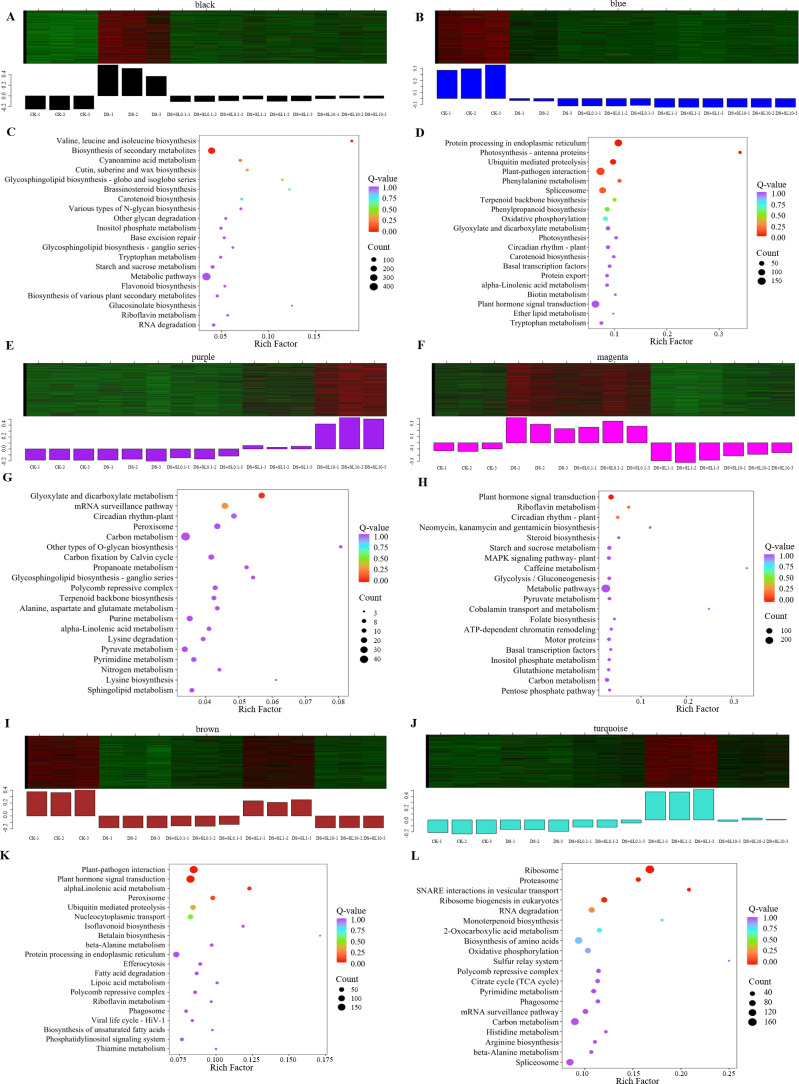
Expression landscapes and KEGG functional enrichment of core WGCNA modules. Panels **(A, B, E, F, I, J)** illustrate gene expression heatmaps and treatment-specific profiles; **(C, D, G, H, K, L)** represent corresponding KEGG pathway enrichment for the black, blue, purple, magenta, brown, and turquoise modules, respectively. Heatmaps are based on Z-score normalized FPKM values.

Based on the systematic analysis of co-expression module expression patterns, the Turquoise module exhibited a highly specific up-regulation under 1 μmol·L^-1^ SL, characterized by a unimodal expression pattern (**Figure 5B**). This module showed significant positive correlations with SD, Fv/Fm, and Ci (**Figure 5C**), and it was extremely significantly enriched in the “Ribosome” pathway (**Figure 6J**). Concurrently, the Brown module exhibited a sharp down-regulation following drought stress but experienced a significant expression rebound under the 1 μmol·L^-1^ SL concentration (**Figure 5B**). This module showed significant positive correlations with PH, SD, SPAD, and Pn (**Figure 5C**), and it was significantly enriched in pathways such as “Plant hormone signal transduction”, “alpha-Linolenic acid metabolism”, and “Peroxisome” (**Figure 6K**).

#### 3.4.2 Identification of hub genes and co-expression networks associated with stress alleviation

**Figure 7:**
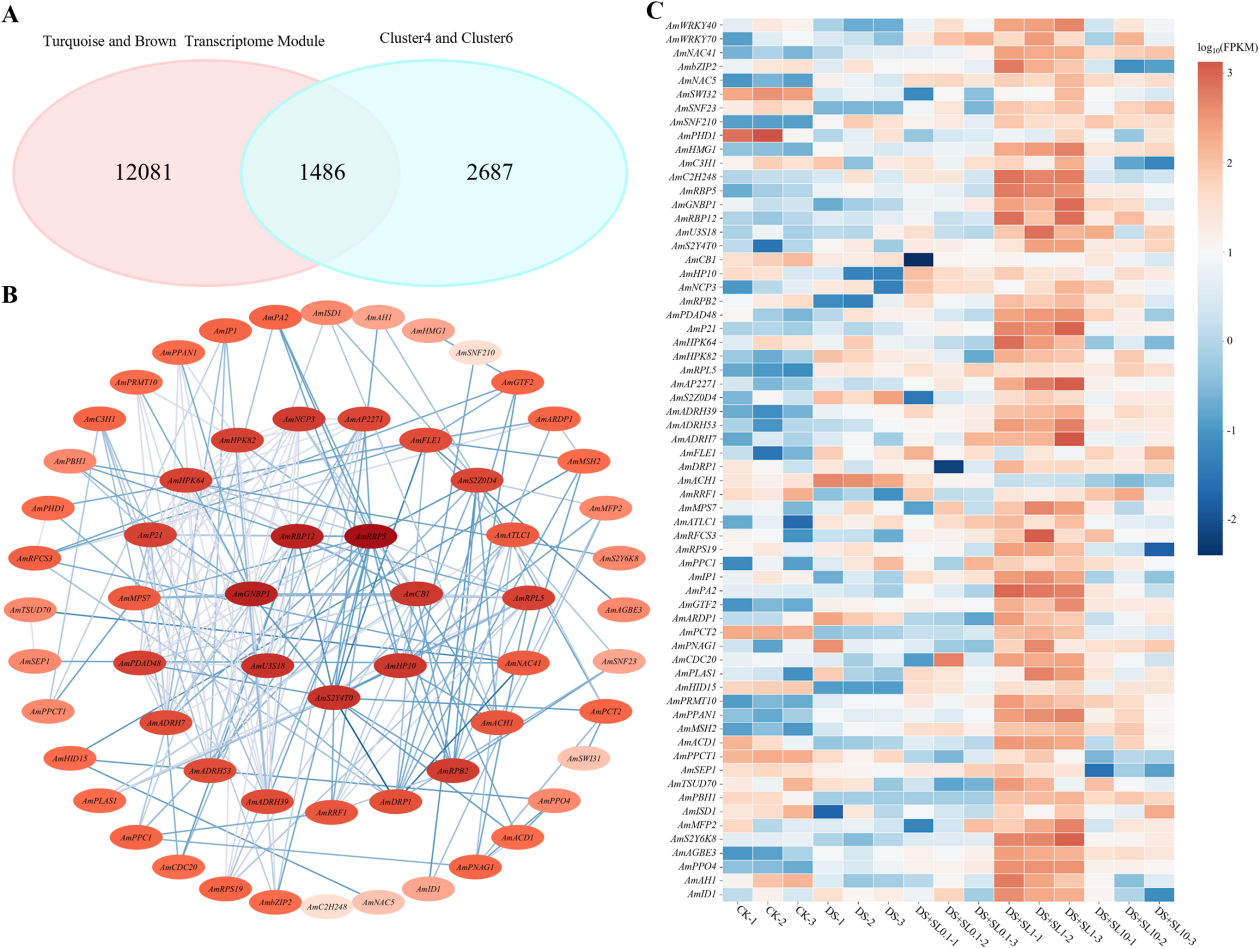
Intersection, co-expression network, and expression profiling of core transcription factors. **(A)** Venn diagram illustrating the intersection of genes in the Turquoise and Brown modules with Clusters 4 and 6; **(B)** Co-expression regulatory network constructed using the top 64 core genes with the highest connectivity scores from the intersected gene set, Nodes are labeled with putative gene symbols (e.g., *AmWRKY40*); **(C)** Heatmap showing the relative expression levels (log_10_FPKM) of the identified core hub genes across the different treatment groups (CK, DS, DS+SL0.1, DS+SL1, and DS+SL10). The detailed annotation and ID mapping are listed in **Supplementary Table 1**.

To identify the hub genes potentially associated with the 1 μmol·L^-1^ SL treatment, this study screened for the intersection of genes within the Turquoise and Brown modules and those significant up-regulation trends in K-means clustering, specifically Clusters 4 and 6. This process identified 1,486 shared differentially expressed genes as shown in **Figure 7A**. Subsequently, protein-protein interaction analysis based on the STRING database initially identified 142 core hub genes. To optimize network visualization and focus on the most tightly co-expressed nodes, the top 64 genes with the highest connectivity scores were selected to construct the core co-expression network presented in **Figure 7B**. As illustrated in the expression heatmap (**Figure 7C**), the expression levels of these hub transcription factors were robustly induced under the 1 μmol·L^-1^ SL treatment, correlating with the optimal physiological recovery.

Within this refined network, 15 key hub transcription factors prominently emerged. These included stress and defense regulatory families, notably WRKY (*AmWRKY40*, *AmWRKY70*), NAC (*AmNAC41*, *AmNAC5*), and bZIP (*AmbZIP2*). The network also encompassed chromatin remodeling-related families including SWI/SNF-SWI3 (*AmSWI31*), SNF2 (*AmSNF23*, *AmSNF210*), and PHD (*AmPHD1*), alongside structural and signaling regulatory families such as HMG (*AmHMG1*), C3H (*AmC3H1*), and C2H2 (*AmC2H248*). These identifiers represent putative orthologs named based on homology alignment with *Cicer arietinum*, as detailed in **Supplementary Table 1**.

These hub transcription factors exhibited tight co-expression with downstream functional genes within the network. To evaluate this association, we profiled the expression dynamics of the remaining functional genes mapped to photosynthesis, antioxidant defense, secondary metabolism, and protein metabolism. The chloroplast-localized gene *AmISD1* and the glutathione metabolism-associated gene *AmID1* were identified as key nodes, and their expression patterns correlated strongly with the recovery in actual photosynthetic parameters and antioxidant capacity. Furthermore, the physiological accumulation of total polysaccharides and total flavonoids was consistent with the co-expression of critical secondary metabolic genes, such as the starch and sucrose metabolism gene *AmAGBE3* and the phenylalanine biosynthesis gene *AmPCT2*. Additionally, the restoration of soluble protein content was closely linked to the up-regulation of genes executing the translation machinery, including the ribosomal proteins *AmRPL5* and *AmRPS19*, alongside the ribosome biogenesis factor *AmGNBP1*.

Collectively, these findings demonstrate that the 1 μmol·L^-1^ SL treatment correlates with the concurrent up-regulation of a complex transcriptional network involving specific transcription factors, secondary metabolic pathways, and ribosome biogenesis. While the expression patterns of the Turquoise and Brown modules are highly correlated with the recovery of physiological traits, the precise mechanistic sequence requires further investigation. The up-regulation of ribosomal and protein processing genes coincides with stress mitigation, but it remains to be determined whether this transcriptional shift acts as the primary driver of functional repair or emerges as a downstream consequence of the overall improved physiological health of the plant.

### 3.5 qRT-PCR validation of hub genes

**Figure 8:**
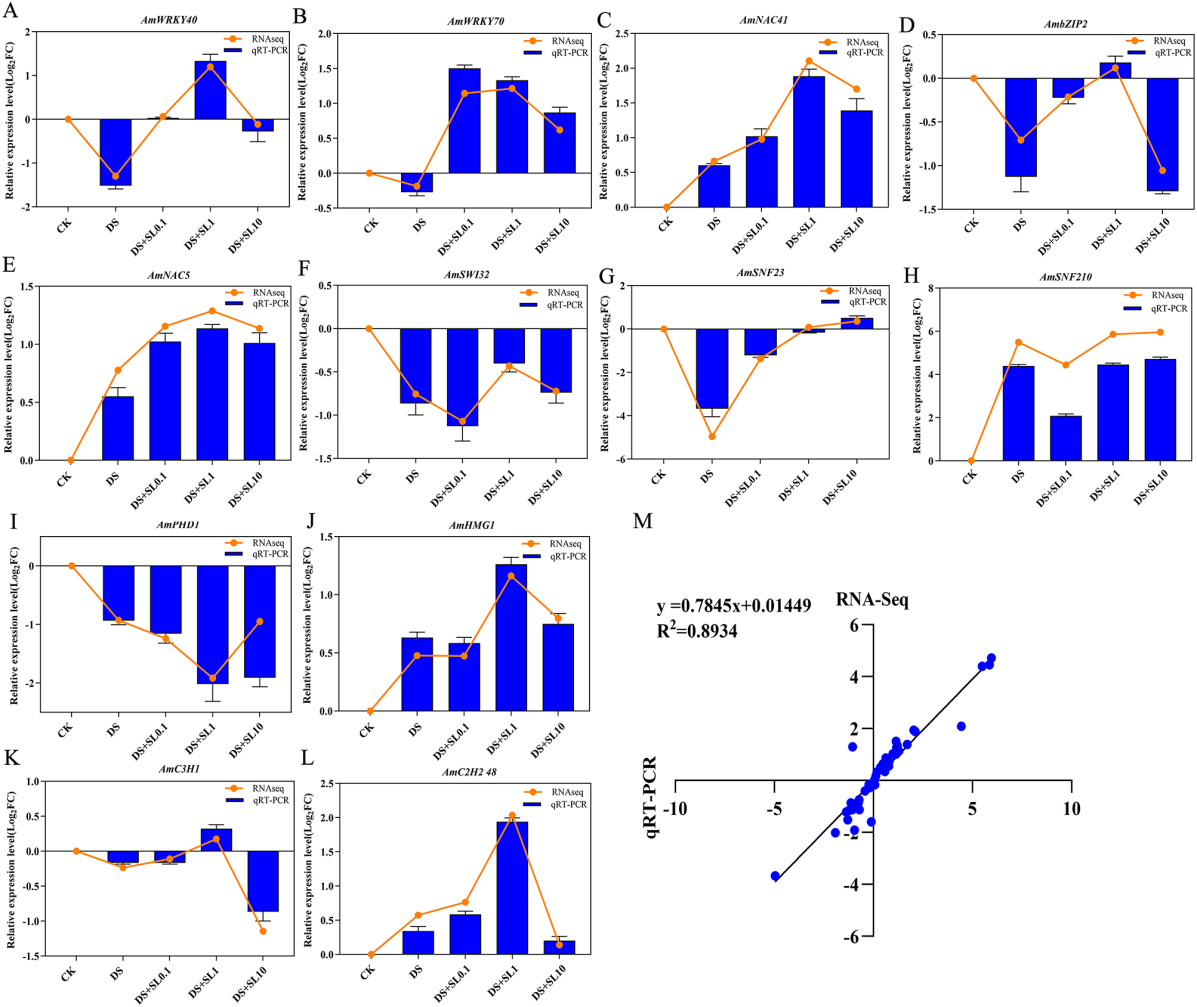
qRT-PCR validation and correlation analysis of core hub genes. **(A–L)** Comparison of log_2_(FoldChange) values between RNA-Seq (orange lines) and qRT-PCR (blue bars) for 12 selected hub genes across different treatments. **(M)** Linear correlation analysis. The scatter plot demonstrates a significant positive correlation (R^2^ = 0.8934) between qRT-PCR and RNA-Seq data. Data are means ± SE (n=3). 18S rRNA was used as the internal control for normalization.

To verify the transcriptomic reliability, 12 identified hub genes (including *AmWRKY40*, *AmNAC41*, and *AmSNF210*) were selected for qRT-PCR analysis. These genes represent the core regulatory nodes of the SL-mediated drought response. The expression patterns observed via qRT-PCR were highly congruent with the RNA-Seq log_2_ (FoldChange) values (**Figure 8**). Correlation analysis yielded a coefficient of R^2^ = 0.8934, confirming the robustness of our WGCNA-based hub gene identification and the overall accuracy of the sequencing data.

## 4 Discussion

Drought stress severely hinders the productivity and medicinal quality of *Astragalus* by inducing oxidative damage, inhibiting photosynthesis, and disrupting the stable accumulation of bioactive secondary metabolites. Understanding the molecular and physiological mechanisms underlying drought tolerance is therefore crucial for improving the resilience and clinical efficacy of this economically vital medicinal plant under increasing climate variability. SL, a novel class of plant hormones, has recently gained significant attention for its protective roles against environmental adversities by regulating antioxidant defenses, protecting chloroplast ultrastructure, and modulating stress-responsive gene expression. To elucidate how SL mitigates drought stress in *Astragalus* and is associated with functional reconstruction, we examined the effects of the synthetic analog rac-GR24 (hereinafter referred to as SL) at three concentrations (0.1, 1, and 10 μmol·L^-1^) under controlled drought conditions, compared with well-watered and drought-only controls. Based on these treatments, comprehensive physiological parameter measurements and WGCNA-based transcriptomic analyses were integrated to identify the core regulatory modules and hub transcription factors involved in the SL-mediated drought response. The findings of this study provide a theoretical framework and potential applications for understanding the molecular logic by which appropriate SL levels promote stress reversal and for optimizing the high-quality cultivation of *Astragalus* in arid environments.

### 4.1 Effects of exogenous SL on plant phenotypic and physiological characteristics under drought stress

Drought stress imposed dual constraints of growth inhibition and oxidative damage on *Astragalus* seedlings. In this study, the observed significant decrease in PH, accompanied by reductions in SD and chlorophyll fluorescence parameters, suggested that water deficit might have restricted cell elongation and impaired Photosystem II activity (Hu et al., 2023; Rashkov et al., 2025; Wang et al., 2025d). The synchronous decline in Pn, SPAD, and Ci indicated that restricted carbon dioxide assimilation and impaired photosynthetic efficiency were the core factors leading to biomass loss. Meanwhile, drought induced severe lipid peroxidation, manifested as a significant increase of 29.13% in MDA content, confirming the occurrence of severe oxidative stress (Kolupaev et al., 2023). Although *Astragalus* initiated a defensive compensatory response by increasing endogenous POD and CAT activities and accumulating TPS and TFC, these defense mechanisms were evidently insufficient to completely prevent oxidative damage under severe drought conditions (Jan et al., 2024).

Exogenous SL significantly alleviated the aforementioned adverse effects and exhibited a significant concentration dependence. In the 0.1 μmol·L^-1^ SL treatment, CAT activity and TFC content reached their peak values. According to prior research, this likely reflects a preliminary activation of the defense system triggered by SL at low dosages, manifested as an enhancement of antioxidant and secondary metabolic defenses (Jan et al., 2022; Liu et al., 2025a). Notably, the 1 μmol·L^-1^ SL treatment was proven to be the most effective concentration for restoring growth and photosynthetic assimilation capacity, as its PH and Gs indices were all superior to other groups, and SPC recovered significantly. This protective effect suggested that 1 μmol·L^-1^ SL might have played an important role in maintaining stomatal opening and protecting protein homeostasis. In contrast, although the 10 μmol·L^-1^ SL treatment reduced MDA to the lowest value and induced massive accumulation of TPS, it led to a sharp decline in SPC. These results implied a dosage trade-off effect, whereby an excessively high concentration of SL might shift from protection to a potential inhibitory effect on protein homeostasis.

In summary, moderate SL supplementation, especially 1 μmol·L^-1^, was associated with enhanced drought resistance in *Astragalus*, alongside strengthened antioxidant defense and preserved photosynthetic function. However, its mitigating efficacy exhibited strict dose dependence, emphasizing the necessity of optimizing application levels in practical applications to avoid potential physiological inhibitory effects such as SPC decline. These findings provided a physiological basis for using SL as a biostimulant to improve the resistance of *Astragalus* in drought environments and offered a theoretical framework for supporting high-quality *Astragalus* production under water-limited conditions.

### 4.2 Key drought stress-responsive hub genes identified by trend clustering and WGCNA analyses

Our integrated analysis, synergizing K-means transcriptional clustering with WGCNA, revealed a complex network of drought-responsive hub genes spanning transcriptional regulation, chromatin remodeling, and metabolic reconstruction. These genes constitute interconnected modules that are closely associated with the physiological resilience of *Astragalus* under drought stress. Notably, the Turquoise module exhibited a distinct unimodal expression pattern characterized by high specific induction under 1 μmol·L^-1^ SL treatment. This module showed significant positive correlations with SD, *Fv*/*Fm*, and Ci, suggesting its potential involvement in the transition of damaged tissues from an emergency defense state to a functional repair state. These correlations imply that the Turquoise and Brown modules may serve as a key regulatory hub associated with this physiological shift.

To precisely identify the regulatory key regulatory candidates, we screened for the intersection between Turquoise and Brown module genes and transcription factors exhibiting significant up-regulation trends specifically within K-means Clusters 4 and 6. This strategy pinpointed 1,486 differentially expressed TFs, and subsequent PPI analysis identified 15 pivotal hub TFs potentially forming a core regulatory cascade associated with SL-induced functional recovery. Among these, members of the WRKY (*AmWRKY40*, *AmWRKY70*), NAC(*AmNAC41*), and bZIP (*AmbZIP2*, *AmNAC5*) families exhibited high connectivity within the co-expression network, suggesting they may function as potential regulatory nodes. Based on their homology to known stress-responsive transcription factors, these WRKY members are hypothesized to participate in the regulation of antioxidant defense, potentially via modulating *SOD* and *APX* activities to scavenge reactive oxygen species (ROS) and maintain cellular redox balance (Nakashima et al., 2025). The NAC hub gene (*AmNAC41*), a homolog of the tomato (*Solanum lycopersicum*) *SlNAC41* (*SlJUB1*), functions as a positive regulator of drought tolerance, balancing growth and defense by inhibiting ROS accumulation (Thirumalaikumar et al., 2018). Similarly, the bZIP family members are implicated in ABA-dependent signaling pathways (Luqman et al., 2023). Consistent with findings in *Cicer arietinum*, these bZIPs likely activate SnRK2 kinases to initiate the transcription of downstream protective genes, such as those encoding LEA proteins and osmoprotectants (Sunkar, 2017).

Beyond transcriptional regulation, hub genes associated with chromatin remodeling and structural signal processing, specifically the SWI/SNF(*AmSWI31*), SNF2(*AmSNF23*, *AmSNF210*), PHD(*AmPHD1*), and C2H2(*AmC2H248*) families, demonstrated high connectivity within the repair network. The SWI3 subunit of the SWI/SNF complex is essential for maintaining complex integrity and remodeling chromatin structure to facilitate the transcription of drought-responsive genes (Jerzmanowski, 2007; Sunkar, 2017). The core regulatory mechanism of SNF2 family genes (*GmCHR* genes) in soybean (*Glycine max*) under drought stress is the dense distribution of stress-responsive elements such as ABRE, MBS, and DRE/CRT in their promoter regions, which enables them to perceive and respond to drought signals and subsequently participate in transcriptional regulation related to drought stress adaptation (Wang et al., 2023). Tomato *SlCHR1* is an ortholog of *AmSNF23*, and its overexpression leads to significant reductions in seedling root length, hypocotyl length, and cotyledon area, resulting in a compact overall plant architecture, a phenomenon that is even more pronounced during growth under drought stress (Folta et al., 2016). Additionally, the PHD finger protein acts as a specific reader of histone methylation, activating drought-resistance genes through epigenetic modifications (Nishanth et al., 2025). Coordinated with these is the C2H2 zinc finger protein, which likely functions to fine-tune the balance between growth and stress responses by activating defense genes while repressing non-essential pathways (Nakashima et al., 2025).

Importantly, these upstream transcriptional regulators do not act in isolation. As depicted in our functional co-expression network (**Figure 8**), hub TFs are tightly linked to downstream structural genes highly associated with these physiological responses. For instance, the robust recovery of antioxidant capacity and photosynthetic efficiency under 1 μmol·L^-1^ SL is strongly associated with the up-regulation of key functional nodes, such as the glutathione metabolism gene *AmID1* and the chloroplast-localized *AmISD1*. Furthermore, the dynamic accumulation of key secondary metabolites (TPS and TFC) aligns with the co-expression of *AmAGBE3* (starch and sucrose metabolism) and *AmPCT2* (phenylalanine biosynthesis). This tight co-expression pattern suggests that hub TFs like *AmWRKY40* and *AmNAC41* are potentially linked to these downstream effectors, with their expression patterns mirroring the targeted mitigation of drought-induced damage.

Collectively, these findings indicate that the SL-mediated drought tolerance in *Astragalus* is closely linked to a highly coordinated transcriptional, epigenetic, and functional network. The concurrent activation of antioxidant defense (WRKY, NAC), ABA signaling (bZIP), and chromatin remodeling (SWI/SNF, PHD), alongside specific downstream metabolic effectors during 1 μmol·L^-1^ SL treatment, was accompanied by an improved protein translation machinery and metabolic networks. Notably, the restoration of soluble protein content (SPC) was closely linked to the up-regulation of genes executing the translation machinery, including the ribosomal proteins *AmRPL5* and *AmRPS19*, alongside the ribosome biogenesis factor *AmGNBP1*. This coordinated transcriptomic shift mirrors the functional improvement and physiological recovery observed under the optimal SL treatment.

### 4.3 Analysis of SL mediated functional reconstruction mechanisms in damaged *Astragalus* tissues based on KEGG enrichment results

Drought stress induces extensive transcriptome reprogramming in plants, initiating with the rapid perception of calcium signaling and ROS waves (Waters et al., 2009). Through kinase cascades such as SnRK2 and MAPK, it activates core transcription factor networks including bZIP, NAC, and WRKY. This network decisively inhibits photosynthesis and growth by downregulating GLK and stabilizing DELLA to conserve energy (Huot et al., 2014; Mahmood et al., 2020), while simultaneously establishing defensive fortifications by activating antioxidant, osmotic adjustment, and autophagy systems (Kim et al., 2022; Del-Saz et al., 2022; Liu et al., 2022; Agbemafle et al., 2023).

In this study, joint analysis based on K-means trend clustering and WGCNA revealed that 1 μmol·L^-1^ SL was specifically associated with the up-regulation of the Turquoise module, which exhibited high enrichment in “Ribosome”, “Ribosome biogenesis in eukaryotes”, and “Proteasome” pathways. This finding aligns with results in wheat (Kolupaev et al., 2023), where SL treatment alleviated stress injury by enhancing the turnover efficiency of core photosynthetic proteins. Accordingly, we hypothesize that the SL-mediated mitigation of stress injury and improvement in growth efficiency are closely associated with a reshaped dynamic balance between protein synthesis and degradation. However, it should be considered that the up-regulation of these ribosomal genes could also be a secondary consequence of the overall improvement in plant physiological status, rather than the primary driver of repair. Furthermore, the significant recovery of the Brown module under 1 μmol·L^-1^ SL treatment further confirmed the restoration of baseline growth and signaling pathways. Although drought stress caused a sharp downregulation of gene expression in this module, intervention with an appropriate amount of SL was successfully accompanied by an expression rebound, which was significantly positively correlated with PH, SD, and Pn. KEGG analysis indicated that the Brown module was significantly enriched in “Plant hormone signal transduction” and “alpha-Linolenic acid metabolism” pathways. Building upon this pathway-level evidence, our expression profiling revealed that this rebound is accompanied by the restored expression of key functional nodes, such as *AmISD1* (chloroplast-localized) and *AmID1* (peroxisome-associated). This suggests that 1 μmol·L^-1^ SL is associated with alleviated stress-induced damage, potentially involving the coordination of hormone and lipid signaling networks, alongside strengthening energy homeostasis and antioxidant defenses.

Moreover, 1 μmol·L^-1^ SL exerted the most significant upregulation effect on Cluster 4 and Cluster 6, which were primarily enriched in “Flavonoid biosynthesis” as well as “Starch and sucrose metabolism” pathways. This pathway-level enrichment is directly corroborated by our functional co-expression network, which highlights the up-regulation of key metabolic nodes such as *AmPCT2* (phenylalanine biosynthesis) and *AmAGBE3* (starch and sucrose metabolism). Based on this combined transcriptomic evidence, it is inferred that while promoting the accumulation of bioactive antioxidants (TFC), 1 μmol·L^-1^ SL was synchronously and putatively associated with carbohydrate mobilization (TPS), providing a continuous energy flow and structural basis for tissue repair. This mechanism is also supported by studies on rice (Jan et al., 2024), where enhancing metabolic flux of carbon allocation toward starch degradation was found to be conducive to osmotic adjustment following drought.

In summary, these results demonstrate that 1 μmol·L^-1^ SL is linked to altered drought resistance metabolic processes of *Astragalus* and coincides with the molecular reconstruction of the protein translation machinery, strengthening hormone signaling and cell membrane stability, and synergistically mobilizing carbohydrate resources for energy supply and secondary metabolism, coinciding with the recovery of photosynthesis and the enhancement of plant growth rate (Dai et al., 2024; Korek et al., 2025).

### 4.4 Mechanistic dissection of the dose dependent effects of SL under drought stress

Our results explicitly demonstrate a biphasic, dose-dependent response of *Astragalus* to exogenous SL under drought stress. While the lowest concentration (0.1 μmol·L^-1^) failed to elicit a sufficient stress-mitigating signal, the 1 μmol·L^-1^ treatment emerged as the optimal dosage, which was accompanied by an effective restoration of efficiency and biomass. This positive mitigation aligns with previous studies in various crops and medicinal plants, where moderate doses of SL or GR24 consistently improved drought tolerance by boosting antioxidant enzyme activities, maintaining stomatal function, and preserving chlorophyll biosynthesis. For instance, similar optimal protective effects of SL have been documented in medicinal woody plants like crab apple (Xu et al., 2023), economic crops such as tea plants (Shen et al., 2025), as well as major staple crops including wheat (Song et al., 2023) and maize (Luqman et al., 2023).

However, a critical difference between our research and many previous reports is our specific focus on the severe physiological penalties induced by the high dosage (10 μmol·L^-1^). Unlike studies that primarily emphasize the protective efficacy of SL, our integrated physiological and transcriptomic results reveal a classic biphasic dose-response pattern that is consistent with a systemic growth-defense trade-off and metabolic resource misallocation (Karasov et al., 2017), rather than simply reflecting an ‘overactive defensive state’.

Under the 10 μmol·L^-1^ treatment, the elevated SL dosage corresponds to a substantial reallocation of metabolic resources. Physiologically, this condition coincides with a marked accumulation of osmotic protectants, evidenced by the total polysaccharide (TPS) content peaking at 5.92 mg·g^-1^ FW (**Figure 1O**). This phenomenon aligns with the theoretical formation of an internal metabolic sink, which may competitively consume carbon skeletons and essential energy currencies (e.g., ATP and NADPH) (Zhou et al., 2023). In our study, this potential resource diversion is accompanied by signatures of suppressed protein translation. Transcriptomic analysis revealed that down-regulated genes under the 10 μmol·L^-1^ treatment were significantly enriched in “Protein processing in endoplasmic reticulum” (**Supplementary Figure 6H**), a pattern that mirrors the sharp decline in overall soluble protein content (SPC) to 36.17 mg·g^-1^ FW (**Figure 1Q**). Furthermore, this condition is associated with disrupted hormonal crosstalk, consistent with reports of intensified ABA-mediated stomatal closure and repressed growth-promoting gibberellin and auxin pathways (Tian et al., 2022).

Crucially, this pronounced reduction in the soluble protein pool provides a plausible context for the unexpected decline in antioxidant enzyme activities observed in our study. Since defensive enzymes such as SOD, POD, and CAT inherently depend on active translation machinery, the diminished SPC pool may limit their *de novo* synthesis. Thus, the significant reduction in SOD, POD, and CAT activities at this dosage (**Figures 1L–N**) might not indicate a failure of stress perception, but rather emerge as a secondary consequence of extreme metabolic trade-offs, where resources are seemingly diverted toward polysaccharide accumulation over functional protein maintenance. Ultimately, this associated disruption of the protein pool and core photosynthetic enzymes (e.g., Rubisco) correlates with secondary oxidative stress, culminating in the observed severe growth penalty. However, as the current model is constructed on correlative profiling, subsequent genetic validations are required to definitively establish the causal sequence of this high-dose penalty.

### 4.5 Molecular regulatory model of *Astragalus* responding to drought stress and SL regulation

**Figure 9:**
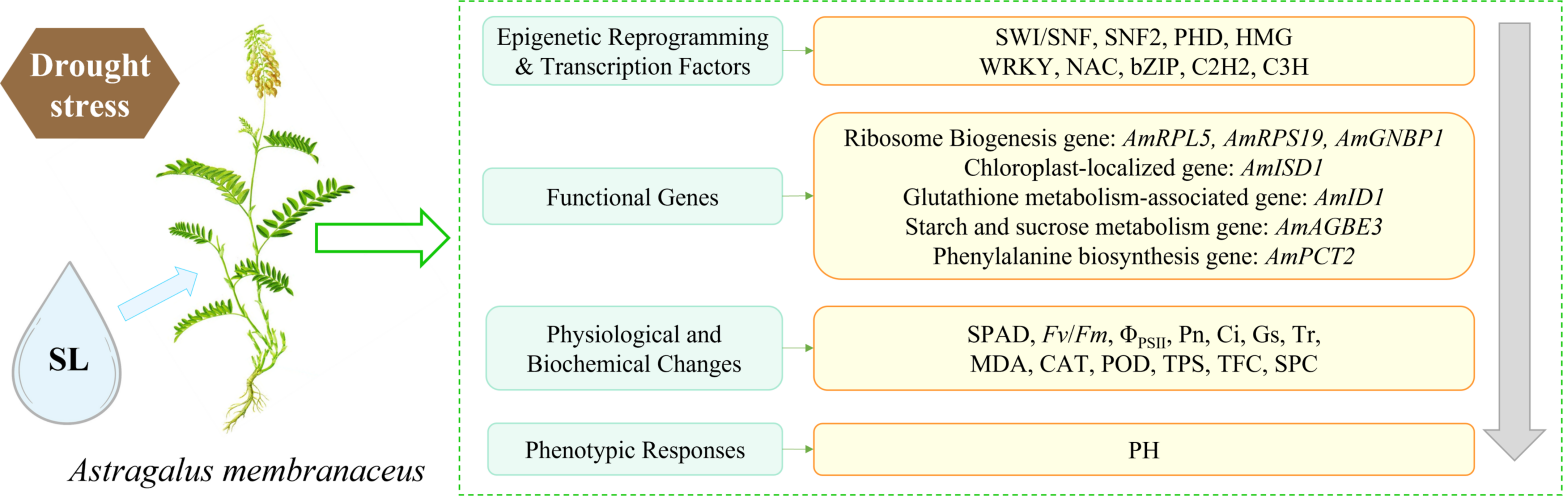
Schematic diagram of the molecular regulatory network of SL alleviating drought damage in *Astragalus*.

Based on the systematic physiological and transcriptomic analyses, we propose a comprehensive molecular regulatory model for SL-mediated drought stress mitigation in *Astragalus* (**Figure 9**). Rather than a definitive causal chain, this model outlines a sequential correlative network from transcriptional responses to phenotypic recovery.

Under severe drought stress, the application of 1 μmol·L^-1^ SL is associated with extensive transcriptomic reprogramming. At the transcriptional level, this treatment coincides with the modulated expression of genes encoding chromatin remodeling complexes (e.g., SWI/SNF, SNF2) and transcription factors (e.g., WRKY, NAC, bZIP). Furthermore, WGCNA reveals that these putative upstream regulators are highly co-expressed with a suite of downstream functional genes. This network includes the up-regulation of ribosome biogenesis genes (e.g., *AmRPL5*, *AmGNBP1*), chloroplast- and peroxisome-associated genes (e.g., *AmISD1*, *AmID1*), and secondary metabolic genes (e.g., *AmAGBE3*, *AmPCT2*).

Third, the expression patterns of these functional genes parallel the observed restoration of cellular physiological and biochemical homeostasis. Specifically, the up-regulation of translation-related genes aligns with the re-establishment of SPC; the induction of chloroplast and antioxidant-related transcripts parallels the recovery of photosynthetic efficiency and the improved scavenging of reactive oxygen species, as evidenced by decreased malondialdehyde and increased CAT and POD activities; and the modulation of metabolic genes is consistent with the accumulation of osmotic protectants, including TPS, and antioxidants like TFC. Importantly, while the up-regulation of ribosomal and functional genes tightly correlates with stress mitigation, the current correlative evidence cannot definitively distinguish whether this transcriptomic shift acts as the primary driver of functional repair or emerges as a secondary consequence of the plant’s overall improved physiological health.

Finally, these physiological improvements are reflected in a macroscopic phenotypic response, corresponding to the recovery of plant growth, specifically plant height. Because the current model is constructed on correlative co-expression networks, subsequent functional validation of these core genes is required to explicitly establish the causal mechanisms underlying SL-mediated drought recovery.

## 5 Conclusion

In this study, a multi-dimensional systematic analysis revealed that Mongolian *Astragalus* exhibits high sensitivity to water deficit. Drought stress is characterized by the activation of the “MAPK signaling pathway” and oxidative stress mechanisms centered on the Black and Magenta modules, which coincides with a decline in photosynthetic system efficiency and severe membrane lipid peroxidation. This constitutes the core physiological logic closely linked to the restricted morphological development and the observed metabolic reconstruction. The intervention of exogenous SL demonstrated a significant concentration-dependent association with stress alleviation. While the 0.1 μmol·L^-1^ concentration yielded weak effects due to insufficient signal intensity, the high concentration of 10 μmol·L^-1^ strengthened osmotic protection associated with the Purple module but coincided with restricted rapid regeneration, paralleling a suppression of soluble protein content and ribosome biogenesis. Under the energy allocation logic that balances plant growth and stress resistance, 1 μmol·L^-1^ was identified as the appropriate concentration correlating with damage alleviation. Its underlying molecular logic is associated with the coordinated up-regulation of the Turquoise and Brown modules, which is characterized by the transcriptomic reconstruction of the protein translation machinery and the restoration of hormone signaling and metabolic homeostasis. This treatment aligns with a hub transcription factor cascade involving WRKY, NAC, and bZIP families that correlates with the up-regulation of “Ribosome biogenesis in eukaryotes” and “Protein processing in endoplasmic reticulum” genes, alongside downstream functional genes (e.g., *AmISD1*, *AmID1*) implicated in photosynthesis and antioxidant defense. This coordinated network correlates with a transition toward a functional repair and growth state, although the up-regulation of ribosomal genes could also emerge as a secondary consequence of improved plant health rather than the primary driver. These findings provide a scientific basis for *Astragalus* cultivation, although the underlying causal mechanisms require further functional validation.

## Data Availability

The datasets presented in this study can be found in online repositories. The names of the repository/repositories and accession number(s) can be found below: https://www.ncbi.nlm.nih.gov/, PRJNA1424176.

## References

[B1] Agbemafle W. Wong M. M. Bassham D. C. (2023). Transcriptional and post-translational regulation of plant autophagy. J. Exp. Bot. 74, 6006–6022. doi: 10.1093/jxb/erad211. PMID: 37358252 PMC10575704

[B2] Ahmad S. Zulfiqar T. Yang H. Ali M. Farooq S. Kouame K. B. (2025). Strigolactones mitigate drought stress in maize by improving chloroplast protection stomatal function and antioxidant defense mechanisms. Ann. Agr Sci. 70, 100389. doi: 10.1016/j.aoas.2025.100389

[B3] Bibi N. Shah M. H. Khan N. Al-Shuraym L. A. Al-Humaid A. I. Ullah I. (2022). Variations in total phenolic, total flavonoid contents, and free radicals’ scavenging potential of onion varieties planted under diverse environmental conditions. Plants 11, 950. doi: 10.3390/plants11070950. PMID: 35406930 PMC9002954

[B4] Cao L. Zhang S. Feng L. Zhang M. Zhang Y. Wang X. (2024). Metabolic pathways regulated by strigolactones foliar spraying enhance osmoregulation and antioxidant defense in drought-prone soybean. BMC Plant Biol. 24, 980. doi: 10.1186/s12870-024-05663-8. PMID: 39420293 PMC11488121

[B5] Chen A. Liu X. Wang Q. Zhang Y. Li Z. (2024a). Strigolactones enhances fodder soybean (Glycine max) adaptation to drought by GmPP2C56 mediating ABA signaling pathway. J. Integr. Agr 23, 100314. doi: 10.1016/j.jia.2024.10.001. PMID: 38826717

[B6] Chen L. Zhang L. G. Li B. H. (2024b). Physiological and transcriptome analyses of Chinese cabbage in response to drought stress. J. Integr. Agr 23, 2255–2269. doi: 10.1016/j.jia.2024.03.067. PMID: 38826717

[B7] Dai B. Wang H. Li W. Zhang X. Liu Y. (2024). Ozone priming enhanced low temperature tolerance of wheat (Triticum aestivum L.) based on physiological, biochemical and transcriptional analyses. Plant Cell Physiol. 65, 1689–1704. doi: 10.1093/pcp/pcae087. PMID: 39096526

[B8] Daszkowska-Golec A. Mehta D. Uhrig R. G. Brąszewska A. Novak O. Fontana I. M. . (2023). Multi-omics insights into the positive role of strigolactone perception in barley drought response. BMC Plant Biol. 23, 445. doi: 10.1186/s12870-023-04450-1. PMID: 37735356 PMC10515045

[B9] Del-Saz N. F. Iglesias-Sanchez A. Alonso-Forn D. López-Gómez M. Palma F . (2022). The lack of alternative oxidase 1a restricts *in vivo* respiratory activity and stress-related metabolism for leaf osmoprotection and redox balancing under sudden acute water and salt stress in Arabidopsis thaliana. Front. Plant Sci. 13, 833113. doi: 10.3389/fpls.2022.833113. PMID: 35656009 PMC9152546

[B10] Dong J. Ding C. Chen H. Fu H. Pei R. Shen F. . (2024). Functions of exogenous strigolactone application and strigolactone biosynthesis genes GhMAX3/GhMAX4b in response to drought tolerance in cotton (Gossypium hirsutum L.). BMC Plant Biol. 24, 1008. doi: 10.1186/s12870-024-05726-w. PMID: 39455926 PMC11515143

[B11] Dong J. Fu H. Wang Z. Zhang L. Liu Z. Hu Y. . (2025). Mechanisms of strigolactone-regulated abiotic stress responses in plants. Plants 14, 2582. doi: 10.3390/plants14162582. PMID: 40872205 PMC12389106

[B12] Dong M. Li J. Yang D. Li M. Wei J . (2023). Biosynthesis and pharmacological activities of flavonoids, triterpene saponins and polysaccharides derived from Astragalus membranaceus. Molecules 28, 5018. doi: 10.3390/molecules28135018. PMID: 37446680 PMC10343288

[B13] Dziwulska-Hunek A. Myśliwa-Kurdziel B. Matwijczuk A. Szymanek M. (2025). A case study in photosynthetic parameters of perennial plants growing in natural conditions. BMC Plant Biol. 25, 1044. doi: 10.1186/s12870-025-07133-1. PMID: 40781277 PMC12333072

[B14] Emami Bistgani Z. Barker A. V. Hashemi M. (2024). Physiology of medicinal and aromatic plants under drought stress. Crop J. 12, 330–339. doi: 10.1016/j.cj.2023.12.003. PMID: 38826717

[B15] Folta A. Bargsten J. W. Bisseling T. Geurts R. (2016). Compact tomato seedlings and plants upon overexpression of a tomato chromatin remodelling ATPase gene. Plant Biotechnol. J. 14, 581–591. doi: 10.1111/pbi.12400. PMID: 25974127 PMC11388966

[B16] Goncharuk E. A. Zubova M. Y. Nechaeva T. L. Kazantseva V. V. Gulevich A. A. Baranova E. N. . (2022). Effects of hydrogen peroxide on *In Vitro* cultures of tea (Camellia sinensis L.) grown in the dark and in the light: Morphology, content of malondialdehyde, and accumulation of various polyphenols. Molecules 27, 6674. doi: 10.3390/molecules27196674. PMID: 36235213 PMC9572957

[B17] Guo X. Yan X. Wang Y. Shi Z. Niu J. Liang J. . (2024). Integrated transcriptomics and metabolomics analysis reveals the effects of cutting on the synthesis of flavonoids and saponins in Chinese herbal medicine Astragalus mongholicus. Metabolites 14, 97. doi: 10.3390/metabo14020097. PMID: 38392989 PMC10891646

[B18] Han X. Yao F. Xue T. T. Wang Z. L. Wang Y. Cao X. . (2022). Sprayed biodegradable liquid film improved the freezing tolerance of cv. Cabernet Sauvignon by up-regulating soluble protein and carbohydrate levels and alleviating oxidative damage. Front. Plant Sci. 13, 1021483. doi: 10.3389/fpls.2022.1021483. PMID: 36388526 PMC9663820

[B19] He Z. Liu X. Qin S. Yang Q. Na J. Xue Z. . (2024). Anticancer mechanism of Astragalus polysaccharide and its application in cancer immunotherapy. Pharmaceuticals 17, 636. doi: 10.3390/ph17050636. PMID: 38794206 PMC11124422

[B20] Hu C. Elias E. Nawrocki W. J. Croce R. (2023). Drought affects both photosystems in Arabidopsis thaliana. New Phytol. 240, 663–675. doi: 10.1111/nph.19171. PMID: 37530066

[B21] Huang Y. Li J. Nong C. Zhang M. Lin Y. Wang Y. . (2024). Piriformospora indica enhances resistance to fusarium wilt in strawberry by increasing the activity of superoxide dismutase, peroxidase, and catalase, while reducing the content of malondialdehyde in the roots. Horticulturae 10, 240. doi: 10.3390/horticulturae10030240. PMID: 30654563

[B22] Huot B. Yao J. Montgomery B. L. He S. Y. (2014). Growth-defense tradeoffs in plants: a balancing act to optimize fitness. Mol. Plant 7, 1267–1287. doi: 10.1093/mp/ssu049. PMID: 24777989 PMC4168297

[B23] Jan R. Asif S. Asaf S. Khan M. A. Kim K. M . (2024). Unveiling the protective role of anthocyanin in rice: insights into drought-induced oxidative stress and metabolic regulation. Front. Plant Sci. 15, 1397817. doi: 10.3389/fpls.2024.1397817. PMID: 38863532 PMC11165195

[B24] Jan R. Khan M. A. Asaf S. . (2022). Drought and UV radiation stress tolerance in rice is improved by overaccumulation of non-enzymatic antioxidant flavonoids. Antioxidants 11, 917. doi: 10.3390/antiox11050917. PMID: 35624781 PMC9137601

[B25] Jariani P. Sabokdast M. Moghadam T. K. Ebrahimi M. A. Asghari B. Penna S. . (2024). Modulation of phytochemical pathways and antioxidant activity in peppermint by salicylic acid and GR24: a molecular approach. Cells 13, 1360. doi: 10.3390/cells13161360. PMID: 39195251 PMC11353152

[B26] Jerzmanowski A. (2007). SWI/SNF chromatin remodeling and linker histones in plants. Biochim. Biophys. Acta 1769, 330–345. doi: 10.1016/j.bbaexp.2006.12.003. PMID: 17292979

[B27] Ji B. Xuan L. Zhang Y. Xu M. Wang Y. Zhang X. . (2023). Advances in biotechnological production and metabolic regulation of Astragalus membranaceus. Plants 12, 1858. doi: 10.3390/plants12091858. PMID: 37176916 PMC10180874

[B28] Jian H. J. Sun H. N. Liu R. R. Zhang J. Li Y. Wang F. . (2022). Construction of drought stress regulation networks in potato based on SMRT and RNA sequencing data. BMC Plant Biol. 22, 381. doi: 10.1186/s12870-022-03758-8. PMID: 35909124 PMC9341072

[B29] Karasov T. L. Chae E. Herman J. J. Bergelson J . (2017). Mechanisms to mitigate the trade-off between growth and defense. Plant Cell 29, 666–680. doi: 10.1105/tpc.16.00931. PMID: 28320784 PMC5435432

[B30] Kim G. Ryu H. Sung J. (2022). Hormonal crosstalk and root suberization for drought stress tolerance in plants. Biomolecules 12, 811. doi: 10.3390/biom12060811. PMID: 35740936 PMC9220869

[B31] Kolupaev Y. E. Yastreb T. O. Ryabchun N. I. Karpets Y. V. Shkliarevskyi M. A . (2023). Response of Triticum aestivum seedlings of different ecological and geographical origin to heat and drought: relationship with resistance to oxidative stress and osmolyte accumulation. Agr Forest 69, 83–99. doi: 10.17707/AgricultForest.69.2.07

[B32] Korek M. Uhrig R. G. Marzec M. (2025). Strigolactone insensitivity affects differential shoot and root transcriptome in barley. J. Appl. Genet. 66, 15–28. doi: 10.1007/s13353-024-00885-w. PMID: 38877382 PMC11762224

[B33] Kwak M. J. Kim Y. I. Lee J. Kim J. Kim E. Kang D. . (2024). Temperature-mediated alterations in the growth, physiology, morphology, and pharmacology of Astragalus membranaceus Bunge: implications for medicinal herb cultivation and therapeutic efficacy. Hortic. Sci. Technol. 42, 433–451. doi: 10.7235/HORT.20240034. PMID: 28809037

[B34] Li H. Jiang X. Mashiguchi K. Yamaguchi S. Lu S . (2024a). Biosynthesis and signal transduction of plant growth regulators and their effects on bioactive compound production in Salvia miltiorrhiza (Danshen). Chin. Med. 19, 108. doi: 10.1186/s13020-024-00971-5. PMID: 39049014 PMC11267865

[B35] Li P. Ren G. Wu F. Zuo X. Bai Z. Liu C. . (2023). Root-specific flavones and critical enzyme genes involved in their synthesis changes due to drought stress on Scutellaria baicalensis. Front. Ecol. Evol. 11, 1113823. doi: 10.3389/fevo.2023.1113823

[B36] Li X. Mu Y. Hua M. Wang J. Zhang X . (2024b). Integrated phenotypic, transcriptomics and metabolomics: growth status and metabolite accumulation pattern of medicinal materials at different harvest periods of Astragalus membranaceus var. mongholicus. BMC Plant Biol. 24, 358. doi: 10.1186/s12870-024-05030-7. PMID: 38698337 PMC11067282

[B37] Li X. Sheng J. Zhang X. Liu Y . (2025). Study on the optimal harvesting age of Astragalus mongholicus under wild-simulated cultivation in Inner Mongolia. Agronomy 15, 269. doi: 10.3390/agronomy15020269. PMID: 30654563

[B38] Liu T. Liu L. Zhou T. Chen Y. Zhou H. Lyu J. . (2025a). Chalcone isomerase gene (OsCHI3) increases rice drought tolerance by scavenging ROS via flavonoid and ABA metabolic pathways. Crop J. 13, 372–384. doi: 10.1016/j.cj.2025.01.019. PMID: 38826717

[B39] Liu Y. Sheng J. Yang J. Li X . (2025b). Metabolic regulation and molecular mechanism of salt stress response in salt-tolerant Astragalus mongholicus. Appl. Sci. 15, 2575. doi: 10.3390/app15052575. PMID: 30654563

[B40] Liu S. Wang L. Zhang Z. Leng Y. Yang F. Xie G. . (2024). The potential of Astragalus polysaccharide for treating diabetes and its action mechanism. Front. Pharmacol. 15, 1339406. doi: 10.3389/fphar.2024.1339406. PMID: 38659573 PMC11039829

[B41] Liu P. Wu X. Gong B. Wang Y. Zhang J. Chen L. . (2022). Review of the mechanisms by which transcription factors and exogenous substances regulate ROS metabolism under abiotic stress. Antioxidants 11, 2106. doi: 10.3390/antiox11112106. PMID: 36358478 PMC9686556

[B42] Love M. I. Huber W. Anders S. (2014). Moderated estimation of fold change and dispersion for RNA-seq data with DESeq2. Genome Biol. 15, 550. doi: 10.1186/s13059-014-0550-8. PMID: 25516281 PMC4302049

[B43] Luqman M. Shahbaz M. Maqsood M. F. Farhat F. Zulfiqar U. Siddiqui M. H. . (2023). Effect of strigolactone on growth, photosynthetic efficiency, antioxidant activity, and osmolytes accumulation in different maize (Zea mays L.) hybrids grown under drought stress. J. Plant Interact. 18, 2262795. doi: 10.1080/15592324.2023.2262795. PMID: 37767863 PMC10730227

[B44] Luqman M. Shahbaz M. Maqsood M. F. . (2023). Effect of strigolactone on growth, photosynthetic efficiency, antioxidant activity, and osmolytes accumulation in different maize (Zea mays L.) hybrids grown under drought stress. Plant Signal. Behav. 18, e2262795. doi: 10.1080/15592324.2023.2262795. PMID: 37767863 PMC10730227

[B45] Lyu M. Liu J. Xu X. Jiang Y. Zhu Z. Ge S. . (2023). Magnesium alleviates aluminum-induced growth inhibition by enhancing antioxidant enzyme activity and carbon-nitrogen metabolism in apple seedlings. Ecotox Environ. Safe 249, 114421. doi: 10.1016/j.ecoenv.2022.114421. PMID: 36529044

[B46] Mahmood T. Khalid S. Abdullah M. Ahmed Z. Shah M. K. N. Ghafoor A. . (2020). Insights into drought stress signaling in plants and the molecular genetic basis of cotton drought tolerance. Cells 9, 105. doi: 10.3390/cells9010105. PMID: 31906215 PMC7016789

[B47] Mohammed E. A. Abdalla I. G. Alfawaz M. A. Mohammed M. A. Al Maiman S. A. Osman M. A. . (2022). Effects of extraction solvents on the total phenolic content, total flavonoid content, and antioxidant activity in the aerial part of root vegetables. Agriculture 12, 1820. doi: 10.3390/agriculture12111820. PMID: 30654563

[B48] Nakashima K. Yamaguchi-Shinozaki K. Shinozaki K. (2025). Transcriptional gene network involved in drought stress response: application for crop breeding in the context of climate change. Phil Trans. R. Soc B. 380, 20240236. doi: 10.1098/rstb.2024.0236. PMID: 40439309 PMC12132078

[B49] Nishanth J. B. Gaddala B. Suji S. Fathima P. R. Premkumar A. Karavadi B. . (2025). Epigenetic mechanisms regulating plant responses to abiotic stress and their role in developing climate resilient crops. Discov. Plants 2, 349. doi: 10.1007/s44372-025-00432-9. PMID: 30311153

[B50] Omoarelojie L. O. Kulkarni M. G. Finnie J. F. Van Staden J . (2019). Strigolactones and their crosstalk with other phytohormones. Ann. Bot. 124, 749–767. doi: 10.1093/aob/mcz105 31190074 PMC6868373

[B51] Rashkov G. D. Stefanov M. A. Borisova P. B. Dobrikova A. G. Apostolova E. L. (2025). The role of the organization of light-harvesting complex II in the drought sensitivity of Pisum sativum L. Int. J. Mol. Sci. 26, 11078. doi: 10.3390/ijms262211078. PMID: 41303560 PMC12652389

[B52] Shen J. Tong M. Yuan Q. Long L. Shi Y . (2025). Exogenous strigolactones modulate antioxidant metabolism via CsD27 to enhance drought tolerance in tea plants. Front. Plant Sci. 16, 1601094. doi: 10.3389/fpls.2025.1601094. PMID: 40546421 PMC12179142

[B53] Sheng F. Yang S. Li M. Wang J. Liu L. Zhang L. . (2024). Research progress on the anti-cancer effects of Astragalus membranaceus saponins and their mechanisms of action. Molecules 29, 3388. doi: 10.3390/molecules29143388. PMID: 39064966 PMC11280308

[B54] Shi Y. Ma P. (2024). Pharmacological effects of Astragalus polysaccharides in treating neurodegenerative diseases. Front. Pharmacol. 15, 1449101. doi: 10.3389/fphar.2024.1449101. PMID: 39156112 PMC11327089

[B55] Shu H. Altaf M. A. Mushtaq N. Fu H. Lu X. Zhu G. . (2023). Physiological and transcriptome analysis of the effects of exogenous strigolactones on drought responses of pepper seedlings. Antioxidants 12, 2019. doi: 10.3390/antiox12122019. PMID: 38136139 PMC10740728

[B56] Song M. Hu N. Zhou S. Xie S. Yang J. Ma W. . (2023). Physiological and RNA-Seq analyses on exogenous strigolactones alleviating drought by improving antioxidation and photosynthesis in wheat (Triticum aestivum L.). Antioxidants 12, 1884. doi: 10.3390/antiox12101884. PMID: 37891963 PMC10604895

[B57] Sunkar R. (Ed.) (2017). “ Plant stress tolerance: methods and protocols,” in Methods mol. Biol New York, NY: Humana Press., 2nd Edn, vol. 1631 . doi: 10.1007/978-1-4939-7136-7

[B58] Thirumalaikumar V. P. Devkar V. Mehterov N. Ali S. Ozgur R. Turkan I. . (2018). NAC transcription factor JUNGBRUNNEN1 enhances drought tolerance in tomato. Plant Biotechnol. J. 16, 354–366. doi: 10.1111/pbi.12776. PMID: 28640975 PMC5787828

[B59] Tian Z. Zhang Y. Zhu L. Jiang B. Wang H. Gao R. . (2022). Strigolactones act downstream of gibberellins to regulate fiber cell elongation and cell wall thickness in cotton (Gossypium hirsutum). Plant Cell 34, 4816–4835. doi: 10.1093/plcell/koac272. PMID: 36040191 PMC9709996

[B60] Upton R. N. Correr F. H. Lile J. Reynolds G. L. Falaschi K. Cook J. P. . (2023). Design, execution, and interpretation of plant RNA-seq analyses. Front. Plant Sci. 14, 1135455. doi: 10.3389/fpls.2023.1135455. PMID: 37457354 PMC10348879

[B61] Wang W. Fang Y. Ju Y. (2024). Strigolactone as a potential target for improving abiotic stress tolerance in horticultural crops. Hortic. Plant J. 10, 1739–1758. doi: 10.1016/j.hpj.2024.03.001. PMID: 38826717

[B62] Wang S. Ma Q. Li C. Zhang S. Liu X. (2025d). Chloroplast responses to drought: integrative mechanisms and mitigation strategies. Int. J. Mol. Sci. 26, 11872. doi: 10.3390/ijms262411872. PMID: 41465300 PMC12732831

[B63] Wang J. Rong Z. Shi W. Zhang Y. Wang W. Zheng Y. . (2025b). Characterization of the WRKY family transcription factors in Astragalus membranaceus and their expression under drought stress. BMC Plant Biol. 25, 593. doi: 10.1186/s12870-025-06592-w. PMID: 40329159 PMC12054254

[B64] Wang L. H. Si C. Yang S. P. Zhong Q. W. Zhang G. N. Zhang H. W. . (2025c). A comprehensive analysis of transcriptome and weighted gene coexpression network (WGCNA) reveals functional genes participating in drought stress response of Jerusalem artichoke (Helianthus tuberosus L.). Plant Growth Regul. 105, 1739–1758. doi: 10.1007/s10725-025-01372-w. PMID: 30311153

[B65] Wang J. Sun Z. Liu H. Yue L. Wang F. Liu S. . (2023). Genome-wide identification and characterization of the soybean Snf2 gene family and expression response to rhizobia. Int. J. Mol. Sci. 24, 7250. doi: 10.3390/ijms24087250. PMID: 37108411 PMC10138738

[B66] Wang Y. F. Tong L. L. Liu H. L. Li B. Zhang R . (2025g). Integrated metabolome and transcriptome analysis of maize roots response to different degrees of drought stress. BMC Plant Biol. 25, 505. doi: 10.1186/s12870-025-06505-x. PMID: 40259225 PMC12013163

[B67] Wang B. Wu B. Ma Y. Liu X. Tao L. Jia L. . (2025a). Astragalus polysaccharides: Structure-immunomodulation relationships, multi-target pharmacological activities, and cutting-edge applications in immune modulation. Front. Immunol. 16, 1714898. doi: 10.3389/fimmu.2025.1714898. PMID: 41383616 PMC12689378

[B68] Wang X. D. Zhang Y. N. Wang X. G. Zhuang Y. Ge S. H. (2025f). Effects of exogenous SL on growth and physiological characteristics of flue-cured tobacco seedlings under different degrees of drought stress. Front. Plant Sci. 15, 1473565. doi: 10.3389/fpls.2024.1473565. PMID: 39902209 PMC11788351

[B69] Wang W. Zhou H. Sen A. Zhang P. Yuan L. Zhou S. . (2025e). Recent advances in the mechanisms and applications of Astragalus polysaccharides in liver cancer treatment: An overview. Molecules 30, 2792. doi: 10.3390/molecules30132792. PMID: 40649307 PMC12250682

[B70] Wani K. Naeem M. Khan M. M. A. Aftab T . (2023). Insights into strigolactone (GR24) mediated regulation of cadmium-induced changes and ROS metabolism in Artemisia annua. J. Hazard Mater. 448, 130899. doi: 10.1016/j.jhazmat.2023.130899. PMID: 36860066

[B71] Waters M. T. Wang P. Korkaric M. Capper R. G. Saunders N. J. Langdale J. A. . (2009). GLK transcription factors coordinate expression of the photosynthetic apparatus in Arabidopsis. Plant Cell 21, 1109–1128. doi: 10.1105/tpc.108.065250. PMID: 19376934 PMC2685620

[B72] Xu J. Li L. Liu Y. Yu Y. Li H. Wang X. . (2023). Molecular and physiological mechanisms of strigolactones-mediated drought stress in crab apple (Malus hupehensis Rehd.) seedlings. Sci. Hortic. 311, 111818. doi: 10.1016/j.scienta.2022.111800. PMID: 38826717

[B73] Zhang K. Chen J. Li J. Liu J. Chen J. Liu X. . (2025). Genome-wide identification of the WRKY transcription factors family and regulation of metabolites under cold stress in Astragalus membranaceus. BMC Plant Biol. 25, 1663. doi: 10.1186/s12870-025-07685-2. PMID: 41315972 PMC12664180

[B74] Zhang S. Qi X. Zhu R. Ye D. Shou M. Peng L. . (2024). Transcriptome analysis of Salvia miltiorrhiza under drought stress. Plants 13, 161. doi: 10.3390/plants13020161. PMID: 38256715 PMC10819027

[B75] Zhang Y. Yang F. Wang Y. Zheng Y. Zhu J. (2023). Effects of acid rain stress on the physiological and biochemical characteristics of three plant species. Forests 14, 1067. doi: 10.3390/f14051067. PMID: 30654563

[B76] Zhi Q. Q. Chen Y. Hu H. Huang W. Q. Bao G. G. Wan X. R. (2024). Physiological and transcriptome analyses reveal tissue-specific responses of Leucaena plants to drought stress. Plant Physiol. Bioch. 214, 108926. doi: 10.1016/j.plaphy.2024.108926. PMID: 38996715

[B77] Zhou J. Liu Y. Li Y. Ling W. Fan X. Feng Q. . (2023). Combined analyses of transcriptome and metabolome reveal the mechanism of exogenous strigolactone regulating the response of elephant grass to drought stress. Front. Plant Sci. 14, 1186718. doi: 10.3389/fpls.2023.1186718. PMID: 37223793 PMC10200884

[B78] Zhu J. Yang L. Cai Y. Zeng X. Zhang Y. Huang W. . (2025). Suitable light intensity stimulated polysaccharide biosynthesis in Bletilla striata pseudobulbs through regulating starch and sucrose metabolism. Plant Stress 18, 101041. doi: 10.1016/j.stress.2025.101041. PMID: 38826717

[B79] Ziaei S. M. Moradi R. Sahabi H. Zaferanieh M. Jafari M . (2025). Effects of seed priming on growth, nutrient uptake, and biochemical responses of Astragalus fasciculifolius Boiss under drought stress. Ann. Agr Sci. 70, 100397. doi: 10.1016/j.aoas.2025.100397. PMID: 38826717

[B80] Zou T. Li Y. Wu Y. Li J. J . (2025). Functional traits of herbaceous plants with ecological restoration potential under drought conditions. Plants 14, 3552. doi: 10.3390/plants14233552. PMID: 41375262 PMC12694109

