# Supplementary material for: WGCNA reveals dose-dependent responses and molecular regulatory networks of strigolactone-mediated drought mitigation in Astragalus membranaceus var. mongholicus
Source: Front Plant Sci. 2026 May 13;17:1811607. doi: 10.3389/fpls.2026.1811607 (PMC13212196; doi:10.3389/fpls.2026.1811607)
Supplement: Supplementary file 1 [file Supplementaryfile1.docx]

Supplementary Material

# Supplementary Figures and Tables

## Supplementary Figures


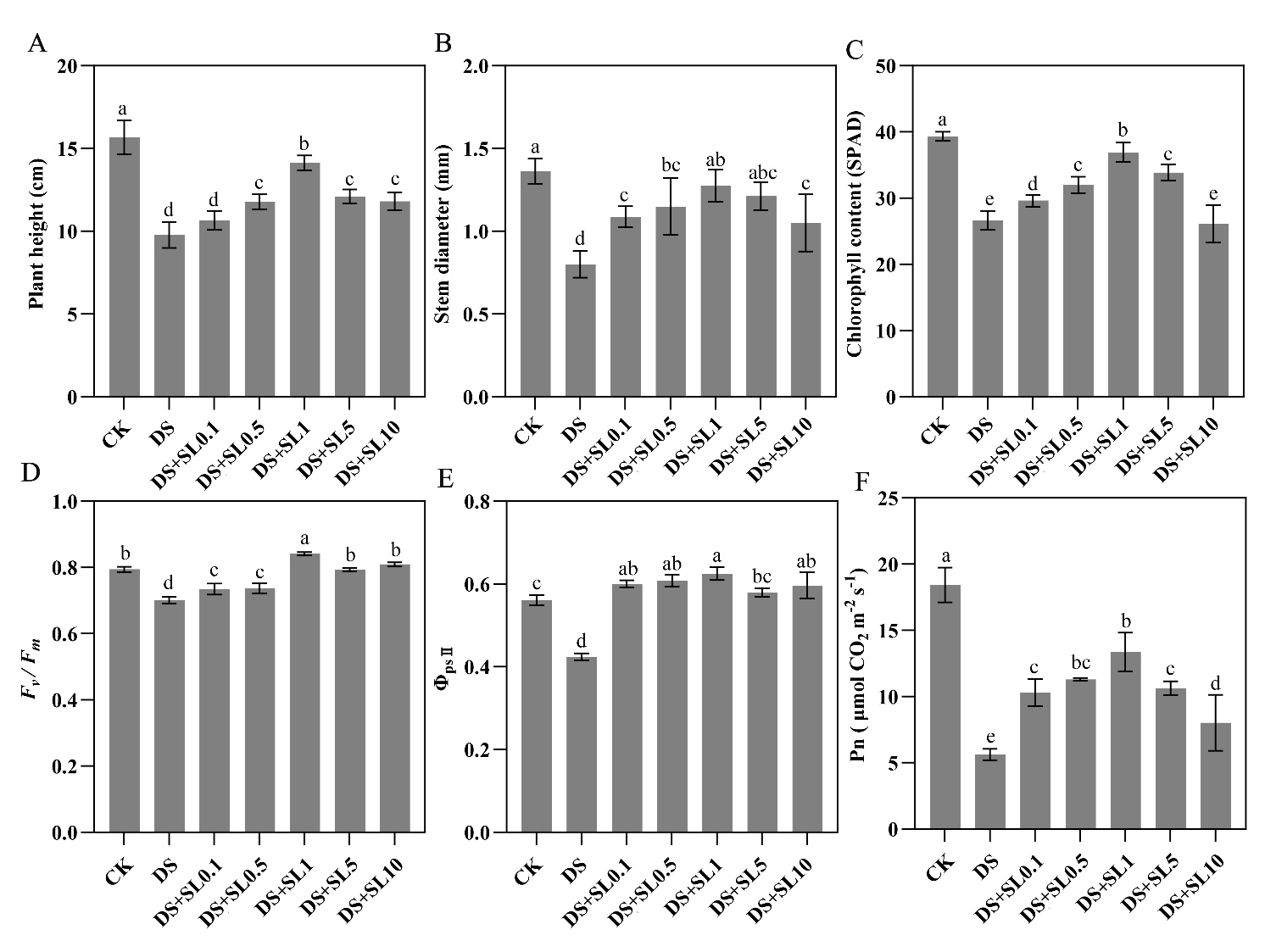


**Supplementary Figure 1.** Preliminary screening of the effects of varying exogenous strigolactone (SL) concentrations on the physiological and photosynthetic traits of *Astragalus* under drought stress. (A) Plant height; (B) Stem diameter; (C) Chlorophyll content (SPAD); (D) Maximum photochemical efficiency of PSII (*Fv/Fm*); (E) Actual photochemical efficiency of PSII (Φ_PSⅡ_); (F) Net photosynthetic rate (Pn). Treatments include CK (well-watered control), DS (drought stress), and DS supplemented with 0.1, 0.5, 1, 5, and 10 μmol·L⁻¹ exogenous SL. Data are presented as means ± SE (n=3). Different lowercase letters indicate significant differences among treatments (LSD test, *p* < 0.05).


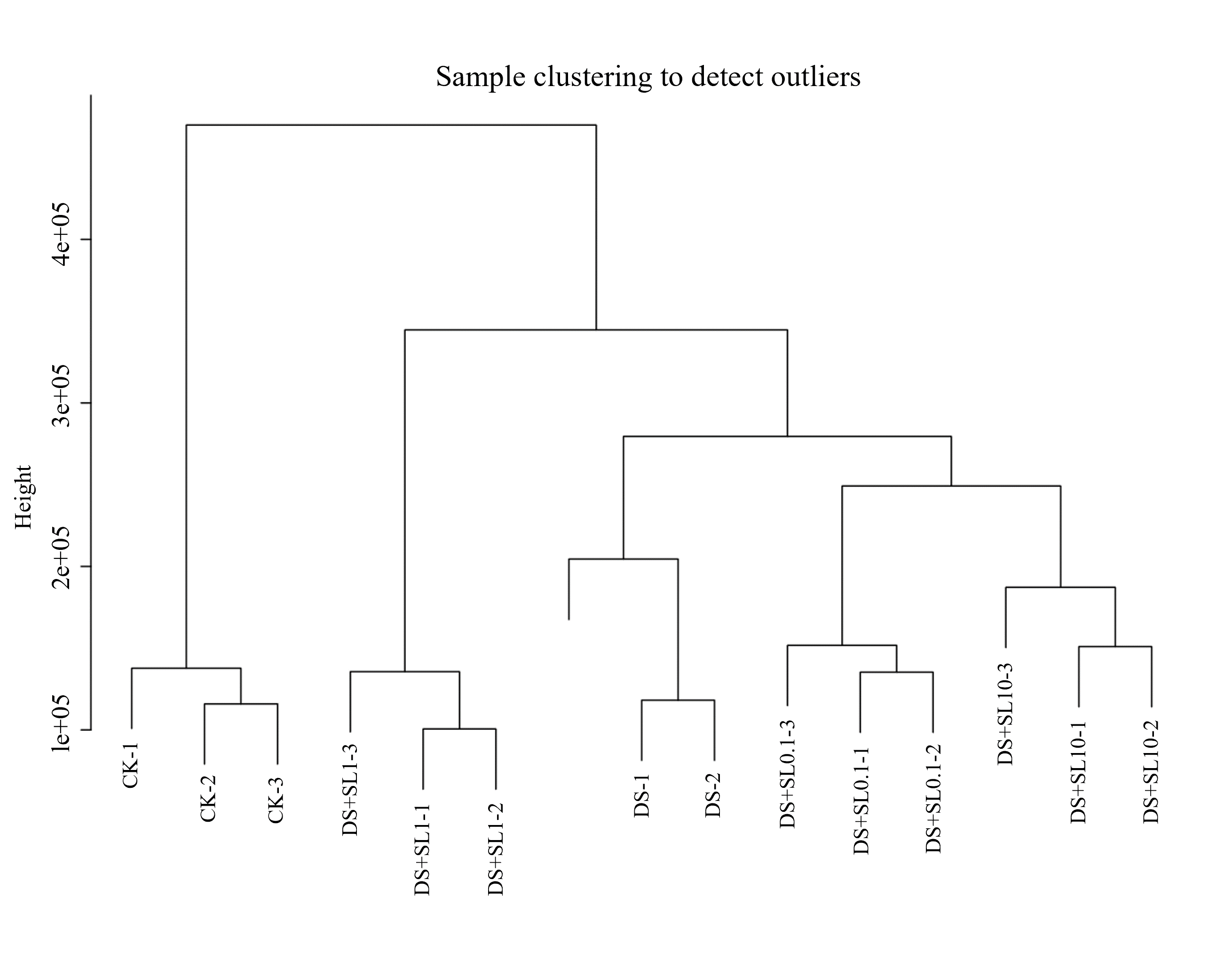


**Supplementary Figure 2.** Sample clustering dendrogram to detect outliers in *Astragalus*. The hierarchical clustering tree displays the relationship among the 15 samples from five treatment groups (CK, DS, DS+SL0.1, DS+SL1, and DS+SL10) based on their overall gene expression profiles. All replicates clustered appropriately within their respective treatment groups, and no outlier samples were identified or removed prior to network construction.


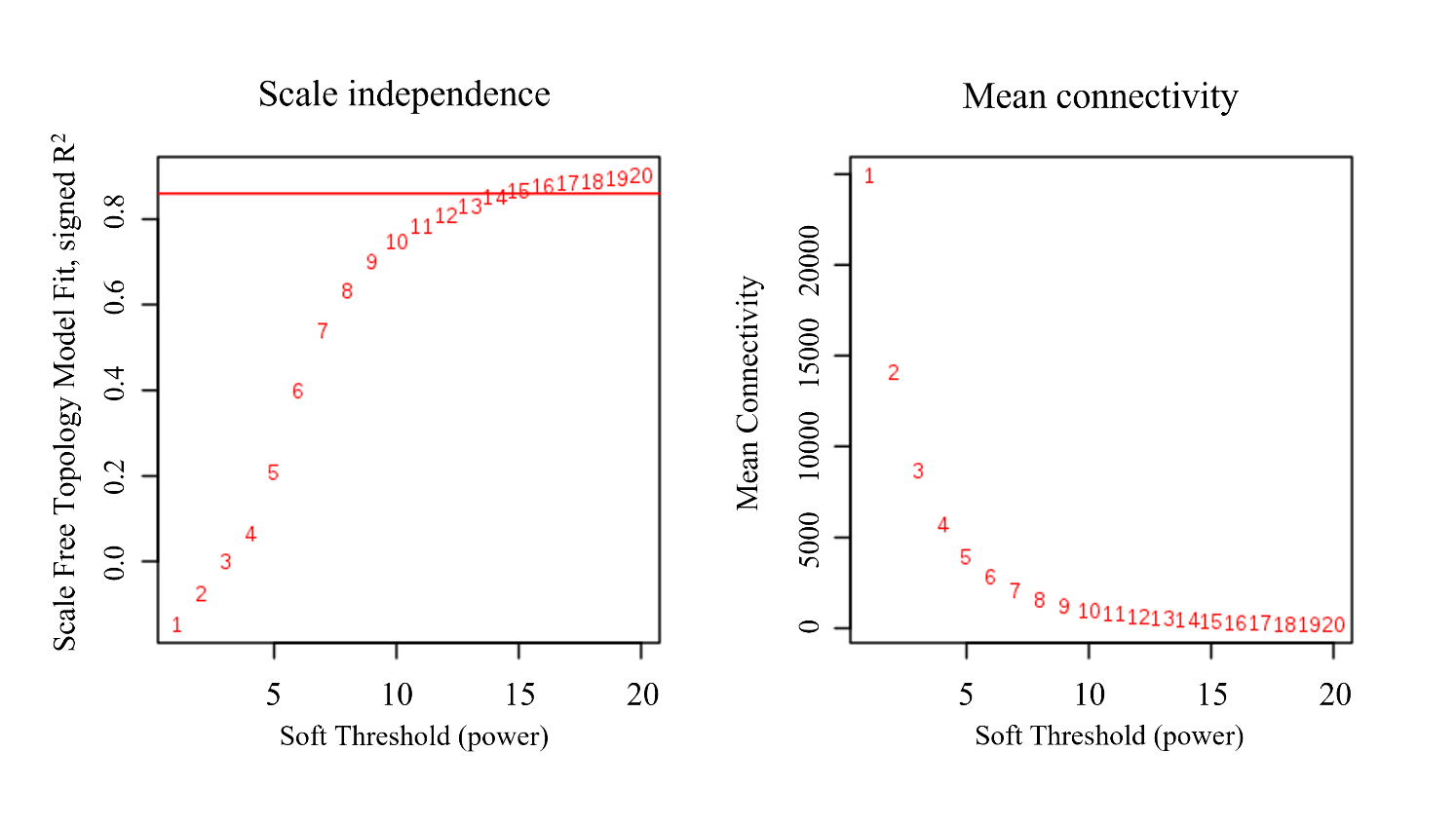


**Supplementary Figure 3.** Determination of the soft-thresholding power in Weighted Gene Co-expression Network Analysis (WGCNA). The plots demonstrate the analysis of network topology for various soft-thresholding powers (β). (Left) The scale-free topology model fit index (R²) as a function of the soft-thresholding power. The red horizontal line indicates the threshold of R² = 0.8, which was successfully achieved at β = 14. (Right) The mean connectivity as a function of the soft-thresholding power, indicating the gradual decrease of connectivity with increasing power.


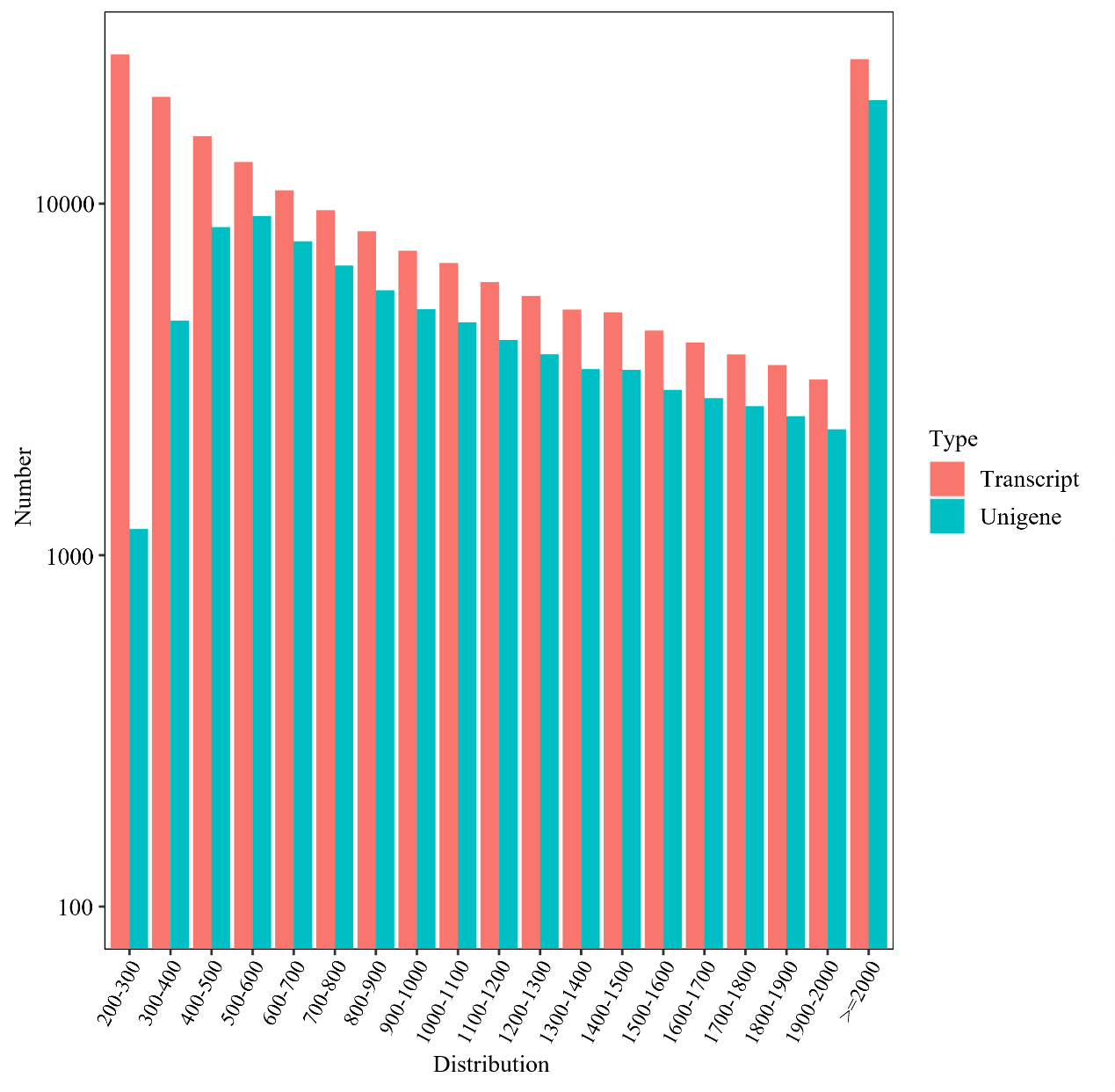


**Supplementary Figure 4.** Length distribution of the assembled transcripts and unigenes in the *Astragalus membranaceus* transcriptome.


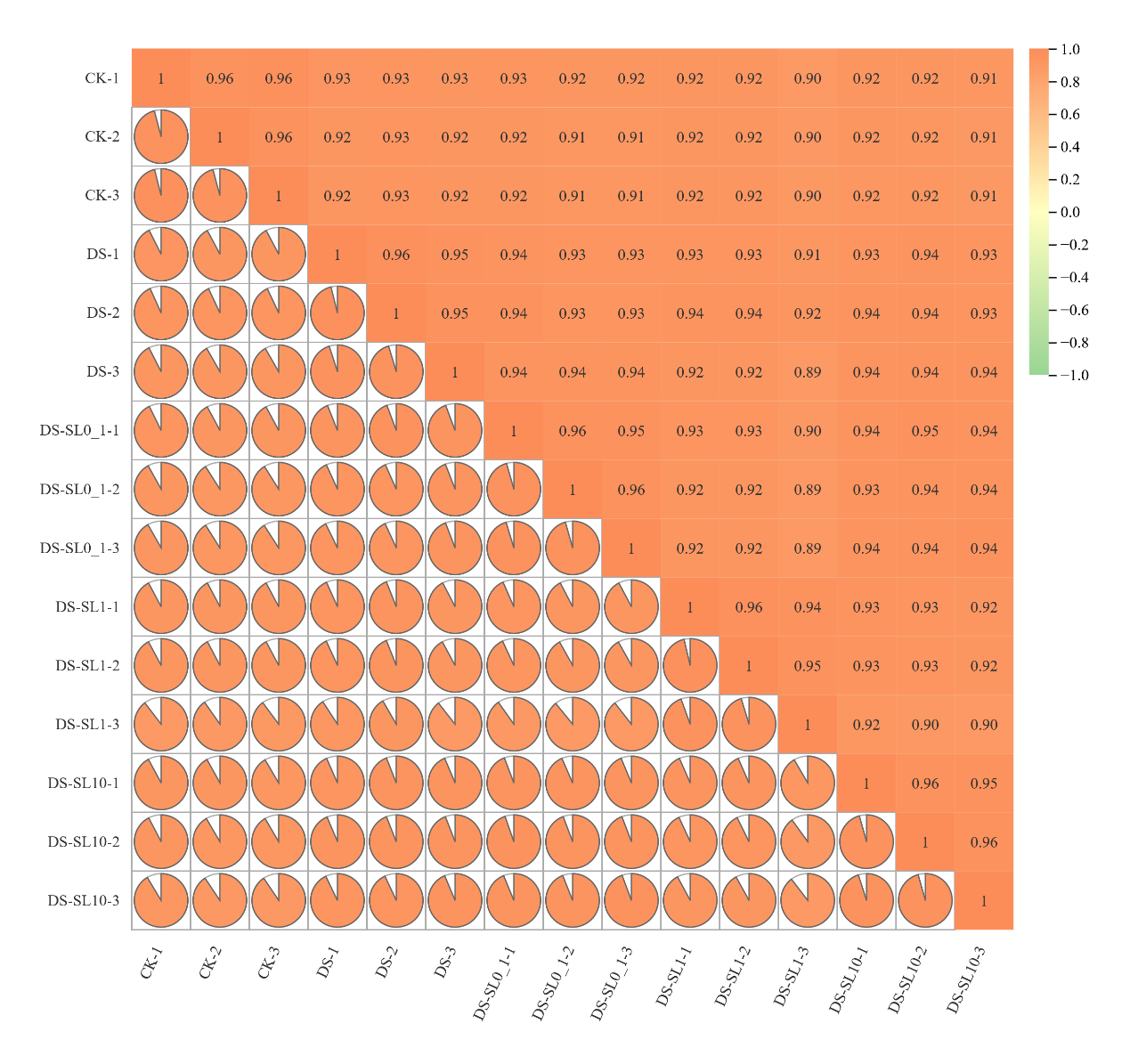


**Supplementary Figure 5.** Pearson correlation heatmap among biological replicates across different treatment groups.


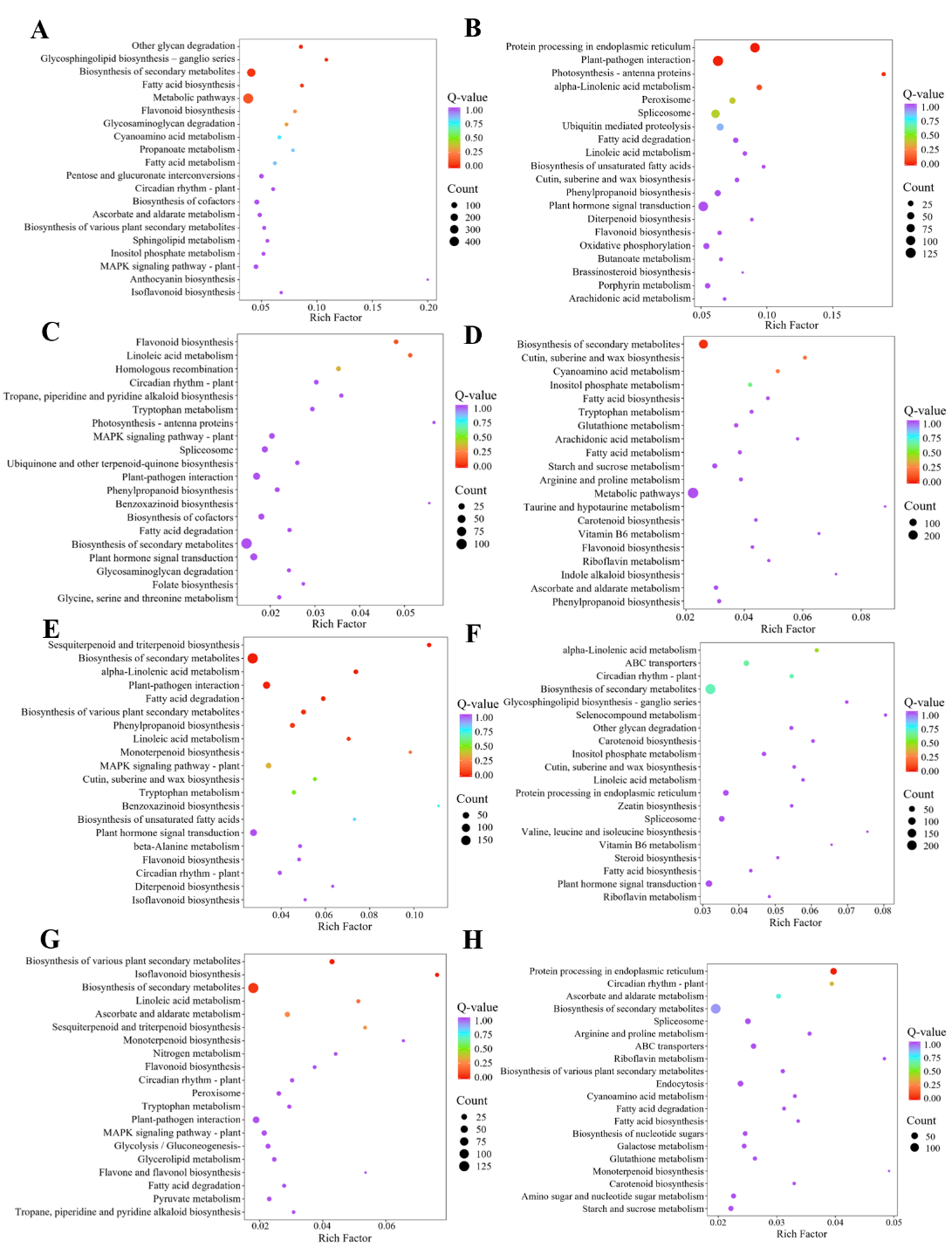


**Supplementary Figure 6.** Scatter plots of KEGG pathway enrichment for DEGs in Astragalus. The significantly enriched pathways for each comparison group are displayed. **(A, B)** Up- and down-regulated genes in CK vs. DS; **(C, D)** DS vs. DS+SL0.1; **(E, F)** DS vs. DS+SL1; (G, H) DS vs. DS+SL10.

## Supplementary Figures

**Supplementary Table 1.** Detailed information of the hub genes identified in the protein-protein interaction (PPI) network.

| Gene Name | Cluster ID  (Transcriptome) | Homolog ID  (Chickpea) | Annotation |
| --- | --- | --- | --- |
| AmWRKY40 | Cluster-43255.3 | A0A1S2Y4T0_CICAR | probable WRKY transcription factor 40 |
| AmWRKY70 | Cluster-32099.3 | A0A1S2XGM5_CICAR | WRKY DNA-binding transcription factor 70-like |
| AmNAC41 | Cluster-42291.0 | A0A1S2YCD9_CICAR | uncharacterized protein NAC41 |
| AmbZIP2 | Cluster-42047.1 | A0A1S3E4H0_CICAR | transcription factor TGA2.2 isoform X1 |
| AmNAC5 | Cluster-29870.0 | A0A1S2Z3B2_CICAR | transcription factor HY5-like |
| AmSWI32 | Cluster-34805.4 | A0A1S2Y4J3_CICAR | lysine-specific histone demethylase 1 homolog 3 isoform X1 |
| AmSNF23 | Cluster-43642.4 | A0A1S2YWE7_CICAR | SNF2 domain-containing protein CLASSY 3 |
| AmSNF210 | Cluster-47216.0 | A0A1S2XQ10_CICAR | probable helicase CHR10 |
| AmPHD1 | Cluster-40660.0 | A0A1S2XZ24_CICAR | BRCT domain-containing protein At4g02110 |
| AmHMG1 | Cluster-20435.3 | A0A3Q7K7G5_CICAR | uncharacterized protein LOC100499992 isoform X1 |
| AmC3H1 | Cluster-38732.4 | A0A1S2XVP7_CICAR | zinc finger CCCH domain-containing protein 1 |
| AmC2H248 | Cluster-21616.0 | A0A1S2Z317_CICAR | uncharacterized protein LOC127093248 |
| AmRBP5 | Cluster-6994.1 | A0A1S2Z316_CICAR | rRNA biogenesis protein RRP5 |
| AmGNBP1 | Cluster-43010.2 | A0A3Q7XJ82_CICAR | guanine nucleotide-binding protein-like NSN1 |
| AmRBP12 | Cluster-33783.2 | A0A1S2XHJ2_CICAR | ribosome biogenesis protein WDR12 homolog |
| AmU3S18 | Cluster-7377.2 | A0A1S2X9I0_CICAR | U3 small nucleolar RNA-associated protein 18 homolog |
| AmS2Y4T0 | Cluster-3169.1 | A0A1S2YEP8_CICAR | uncharacterized protein LOC101498297 |
| AmCB1 | Cluster-38253.0 | A0A1S3E1K0_CICAR | calmodulin binding |
| AmHP10 | Cluster-5832.9 | A0A1S2XT22_CICAR | hypothetical protein TSUD_338710 |
| AmNCP3 | Cluster-34940.0 | A0A1S2XRK2_CICAR | LOW QUALITY PROTEIN: nucleolar complex protein 3 homolog |
| AmRPB2 | Cluster-41676.0 | A0A1S2Y4F4_CICAR | DNA-directed RNA polymerase II subunit RPB2 |
| AmPDAD48 | Cluster-35206.3 | A0A1S2YFL4_CICAR | putative DEAD-box ATP-dependent RNA helicase 48, variant 3 |
| AmP21 | Cluster-32048.0 | A0A1S2XR58_CICAR | p21-activated protein kinase-interacting protein 1-like |
| AmHPK64 | Cluster-40159.3 | A0A3Q7YE57_CICAR | hypothetical protein KIW84_031164 |
| AmHPK82 | Cluster-41036.3 | A0A1S2XVN0_CICAR | hypothetical protein KIW84_063782 |
| AmRPL5 | Cluster-45475.0 | A0A1S2Y7T2_CICAR | 60S ribosomal protein L5-like |
| AmAP2271 | Cluster-26287.0 | A0A1S2XAR5_CICAR | U3 snoRNP-associated protein-like EMB2271 |
| AmS2Z0D4 | Cluster-38223.2 | A0A1S2Z0D4_CICAR | uncharacterized protein LOC101509479 |
| AmADRH39 | Cluster-42699.4 | A0A8B8JUK9_ABRPR | DEAD-box ATP-dependent RNA helicase 39 isoform X1 |
| AmADRH53 | Cluster-34382.0 | A0A1S2XLQ9_CICAR | DEAD-box ATP-dependent RNA helicase 53, mitochondrial-like |
| AmADRH7 | Cluster-42354.0 | A0A1S2YKT4_CICAR | DEAD-box ATP-dependent RNA helicase 7-like |
| AmFLE1 | Cluster-39095.2 | A0A1S2XX27_CICAR | flap endonuclease 1 isoform X1 |
| AmDRP1 | Cluster-36734.1 | A0A1S2Z4A0_CICAR | DNA repair protein UVH3 isoform X1 |
| AmACH1 | Cluster-34604.2 | A0A1S2YZ27_CICAR | aconitate hydratase 1 |
| AmRRF1 | Cluster-35951.1 | A0A1S2YWI6_CICAR | ribosome-recycling factor-like isoform X1 |
| AmMPS7 | Cluster-44583.2 | A0A1S2XVR8_CICAR | multisubstrate pseudouridine synthase 7 |
| AmATLC1 | Cluster-41072.8 | A0A1S2Z0Z5_CICAR | arginine--tRNA ligase, cytoplasmic-like isoform X1 |
| AmRFCS3 | Cluster-37738.1 | A0A1S3E4R2_CICAR | replication factor C subunit 3-like |
| AmRPS19 | Cluster-25827.3 | A0A1S2YWL1_CICAR | 40S ribosomal protein S19-3 |
| AmPPC1 | Cluster-2482.13 | A0A1S2Y5X0_CICAR | phosphoserine phosphatase, chloroplastic |
| AmIP1 | Cluster-39993.2 | A0A1S2Y0Q0_CICAR | PRKR-interacting protein 1 |
| AmPA2 | Cluster-31279.0 | A0A1S2YSZ6_CICAR | phosphoserine aminotransferase 2, chloroplastic-like |
| AmGTF2 | Cluster-32649.0 | A0A1S2XRG4_CICAR | general transcription factor IIH subunit 2 isoform X1 |
| AmARDP1 | Cluster-1983.2 | A0A1S2XSF1_CICAR | BRCA1-associated RING domain protein 1 isoform X1 |
| AmPCT2 | Cluster-35262.1 | A0A1S2YLL6_CICAR | 3-phosphoshikimate 1-carboxyvinyltransferase 2 |
| AmPNAG1 | Cluster-38672.0 | A0A1S2Y3C1_CICAR | probable N-acetyl-gamma-glutamyl-phosphate reductase, chloroplastic |
| AmCDC20 | Cluster-16264.2 | A0A1S2XTG0_CICAR | cell division cycle 20.2, cofactor of APC complex-like |
| AmPLAS1 | Cluster-32123.10 | A0A1S2XBA8_CICAR | phenylalanine--tRNA ligase alpha subunit, cytoplasmic-like |
| AmHID15 | Cluster-46908.3 | A0A1S2YR24_CICAR | histone deacetylase 15 |
| AmPRMT10 | Cluster-18136.0 | A0A1S2Z5U7_CICAR | protein arginine N-methyltransferase PRMT10 |
| AmPPAN1 | Cluster-23153.2 | A0A1S2Z124_CICAR | probable protein arginine N-methyltransferase 1.2 |
| AmMSH2 | Cluster-40714.0 | A0A1S2YR17_CICAR | DNA mismatch repair protein MSH2 |
| AmACD1 | Cluster-39957.1 | A0A1S2YY53_CICAR | acetylornithine deacetylase |
| AmPPCT1 | Cluster-33199.1 | A0A1S2Y7T0_CICAR | peptidyl-prolyl cis-trans isomerase |
| AmSEP1 | Cluster-47343.3 | A0A1S2YWT5_CICAR | seed protein |
| AmTSUD70 | Cluster-35809.7 | A0A1S3E3Z2_CICAR | hypothetical protein TSUD_44670 |
| AmPBH1 | Cluster-48090.2 | A0A1S2YYI5_CICAR | protein BCCIP homolog |
| AmISD1 | Cluster-45012.1 | A0A1S2XM64_CICAR | isocitrate dehydrogenase |
| AmMFP2 | Cluster-37241.4 | A0A1S2YQ71_CICAR | peroxisomal fatty acid beta-oxidation multifunctional protein MFP2 |
| AmS2Y6K8 | Cluster-47160.3 | A0A1S2Y6K8_CICAR | uncharacterized protein LOC101490399 |
| AmAGBE3 | Cluster-47473.0 | A0A1S2Z2J7_CICAR | 1,4-alpha-glucan-branching enzyme 3, chloroplastic/amyloplastic-like |
| AmPPO4 | Cluster-3391.3 | A0A1S2YEI3_CICAR | probable polyamine oxidase 4 |
| AmAH1 | Cluster-34604.6 | A0A1S2YZ27_CICAR | aconitate hydratase 1 |
| AmID1 | Cluster-45012.4 | A0A1S2XM64_CICAR | isocitrate dehydrogenase |

**Supplementary Table 2.** List of primers used for qRT-PCR validation.

| Gene Name | Cluster ID | Primer Name | Sequence (5′→3′) |
| --- | --- | --- | --- |
| 18S rRNA | - | Am.18S rRNA-F | TCAACCATAAACGATGCCGACC |
|  |  | Am.18S rRNA-R | TTTCAGCCTTGCGACCATACTCC |
| AmWRKY40 | Cluster-43255.3 | AmWRKY40-F | CAACAGTGTGGCTATGACAGG |
|  |  | AmWRKY40-R | TTATGTTGCCGGTGATGGTG |
| AmWRKY70 | Cluster-32099.3 | AmWRKY70-F | TCGGCAAAATGGTCATCGTG |
|  |  | AmWRKY70-R | CAGTTCCACCACCACTTCTTAC |
| AmNAC41 | Cluster-42291.0 | AmNAC41-F | TGTGTGTGAATGGGTTCTGC |
|  |  | AmNAC41-R | TCGGAATCGGAACCCTAAAGG |
| AmbZIP2 | Cluster-42047.1 | AmbZIP2-F | TCAATTGGAACCCCTCACAGAG |
|  |  | AmbZIP2-R | TGTTGCAATGCTTCCATGCC |
| AmNAC5 | Cluster-29870.0 | AmNAC5-F | AAAACACCATGCTCCGCAAG |
|  |  | AmNAC5-R | CATCAACTTTGGGCCTCGTATTC |
| AmSWI32 | Cluster-34805.4 | AmSWI32-F | AATTGGAGCAACTGCACGAG |
|  |  | AmSWI32-R | TTCGTCTTCCAATGCTGTCG |
| AmSNF23 | Cluster-43642.4 | AmSNF23-F | ATTGTCAGGCGCATTCAGAG |
|  |  | AmSNF23-R | ACCTTGCTTGTCATCTCACG |
| AmSNF210 | Cluster-47216.0 | AmSNF210-F | TTTTGAGCGGCTGAGAATGC |
|  |  | AmSNF210-R | TACAACCCAAGGCGTACAGTC |
| AmPHD1 | Cluster-40660.0 | AmPHD1-F | AGCGCCGTAAATGAATGTGC |
|  |  | AmPHD1-R | TTCCCTCACCTGCCTTTTCTAC |
| AmHMG1 | Cluster-20435.3 | AmHMG1-F | TTCCATTTCTCACCGCAAGC |
|  |  | AmHMG1-R | ACAAATCTGTCGCCACTGTG |
| AmC3H1 | Cluster-38732.4 | AmC3H1-F | GTTGCTTTGCTATCGTGCTG |
|  |  | AmC3H1-R | TTCTTTTCCAGCGGCTCTTC |
| AmC2H248 | Cluster-21616.0 | AmC2H248-F | AATCGTTTGTTGCCGAGTCG |
|  |  | AmC2H248-R | CCATTCTGAAACCTGGCTCAAC |

**Supplementary Table 3.** Statistics of the transcriptomic data and quality evaluation for the five treatment groups.

| **Sample** | **Group** | **Raw Reads** | **Raw Base(G)** | **Clean Reads** | **Clean Base(G)** | **Error Rate(%)** | **Q20(%)** | **Q30(%)** | **GC Content(%)** |
| --- | --- | --- | --- | --- | --- | --- | --- | --- | --- |
| CK-1 | CK | 53975272 | 8.1 | 53416958 | 8.01 | 0.02 | 99.22 | 97.05 | 44.05 |
| CK-2 | CK | 50955166 | 7.64 | 50391606 | 7.56 | 0.02 | 99.26 | 97.2 | 43.89 |
| CK-3 | CK | 52132604 | 7.82 | 51507418 | 7.73 | 0.02 | 99.26 | 97.21 | 43.83 |
| DS-1 | DS | 49270094 | 7.39 | 48745192 | 7.31 | 0.02 | 99.23 | 97.1 | 44.09 |
| DS-2 | DS | 58229438 | 8.73 | 57549704 | 8.63 | 0.02 | 99.24 | 97.15 | 44.01 |
| DS-3 | DS | 48801386 | 7.32 | 48137522 | 7.22 | 0.02 | 99.18 | 96.92 | 44.76 |
| DS+SL  0.1-1 | DS+SL0.1 | 55755296 | 8.36 | 55087414 | 8.26 | 0.02 | 99.17 | 96.89 | 44.16 |
| DS+SL  0.1-2 | DS+SL0.1 | 47951058 | 7.19 | 47429254 | 7.11 | 0.02 | 99.17 | 96.88 | 44.42 |
| DS+SL  0.1-3 | DS+SL0.1 | 54254566 | 8.14 | 53585122 | 8.04 | 0.02 | 99.1 | 96.65 | 44.4 |
| DS+SL  1-1 | DS+SL1 | 50845898 | 7.63 | 50238596 | 7.54 | 0.02 | 99.27 | 97.26 | 43.82 |
| DS+SL  1-2 | DS+SL1 | 56081320 | 8.41 | 55499562 | 8.32 | 0.02 | 99.42 | 97.8 | 43.84 |
| DS+SL  1-3 | DS+SL1 | 53311546 | 8 | 52687190 | 7.9 | 0.02 | 99.29 | 97.33 | 43.48 |
| DS+SL  10-1 | DS+SL10 | 59009560 | 8.85 | 58318776 | 8.75 | 0.02 | 99.25 | 97.16 | 44.08 |
| DS+SL  10-2 | DS+SL10 | 49506774 | 7.43 | 48946624 | 7.34 | 0.02 | 99.19 | 96.97 | 44.24 |
| DS+SL  10-3 | DS+SL10 | 54940536 | 8.24 | 54302716 | 8.15 | 0.02 | 99.16 | 96.86 | 44.48 |
